# Supplementary material for: Mutations outside the MR1 antigen binding groove differentially inhibit presentation of exogenous antigens
Source: J Biol Chem. 2026 Feb 26;302(4):111317. doi: 10.1016/j.jbc.2026.111317 (PMC13022675; doi:10.1016/j.jbc.2026.111317)
Supplement: Supplementary Material 1 [file mmc1.pdf]

Supporting Information for

Mutations outside the MR1 antigen binding groove differentially inhibit presentation of exogenous antigens

Corinna A. Kulicke, Chance Lemon, Jason R. Krawic, Luisa Maria Nieto Ramirez, Se-Jin Kim, Gitanjali Narayanan, Fikadu G. Tafesse, William H. Hildebrand, Karen M. Dobos, David M. Lewinsohn

Figures S1 – S17

Tables S1 – S10

SI References

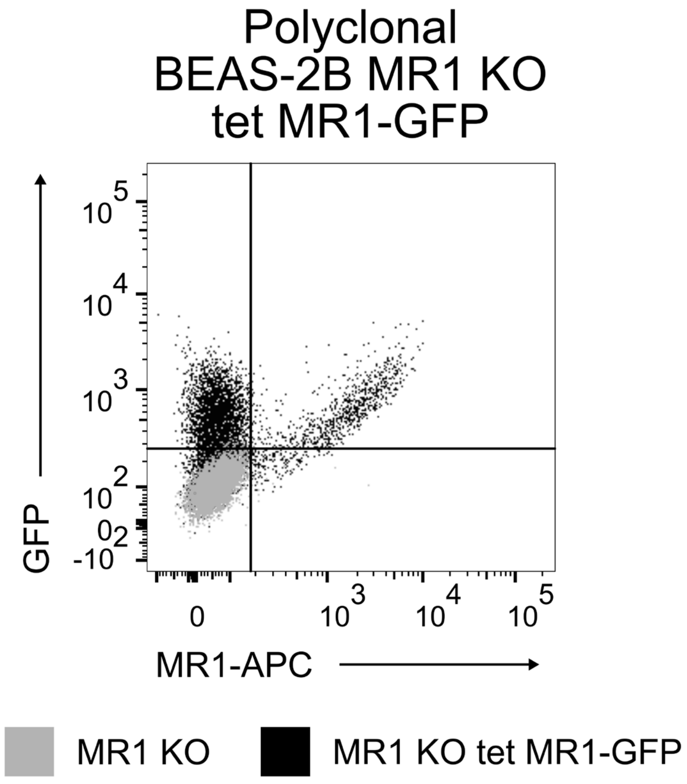

Figure S1: A polyclonal reconstituted cell line expresses heterogenous levels of MR1 at the cell surface. Polyclonal BEAS-2B MR1 KO tet MR1-GFP cells were induced to express MR1-GFP with 2 µg/ml doxycycline (dox) overnight and stained for MR1 surface expression. The BEAS-2B MR1 KO parent cell line is shown for comparison. Data are representative of three independent experiments. KO = knock out; tet = tetracycline.

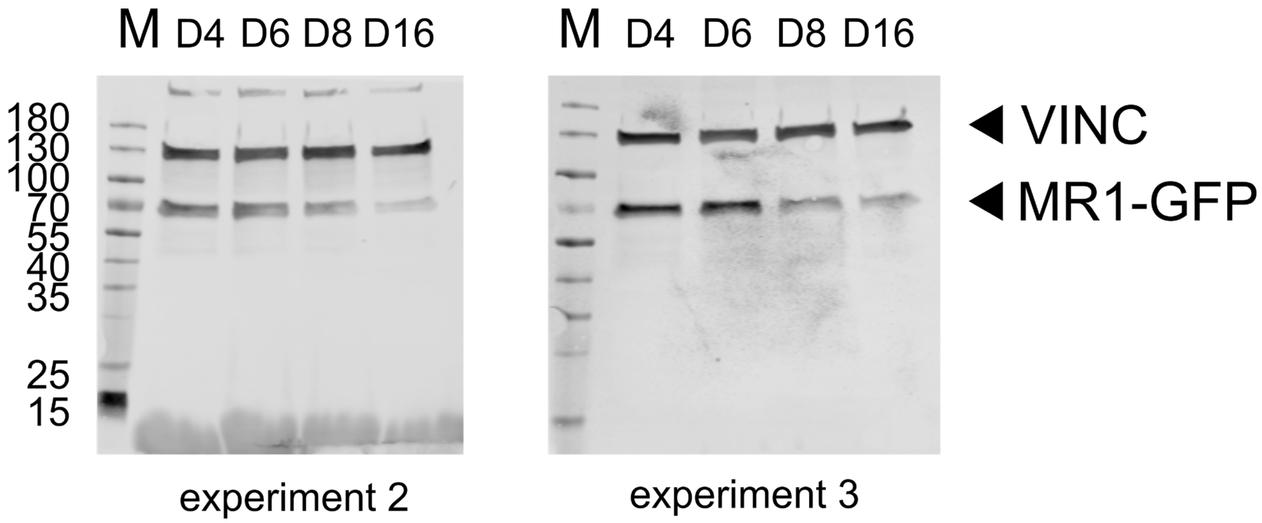

Figure S2: Repeat experiments of the Western blot shown in Figure 1C. WB analysis of MR1-GFP expression as in Figure 1C. Whole cell lysates from cell lines induced with 2 µg/ml doxycycline overnight were analyzed for expression of MR1-GFP and loading control Vinculin (VINC) by WB. Primary antibodies were from different species and detected in parallel with species-specific secondary antibodies conjugated to distinct IRDyes and both channels exported as greyscale. Molecular weight markers are indicated in kDa on the left. M = marker.

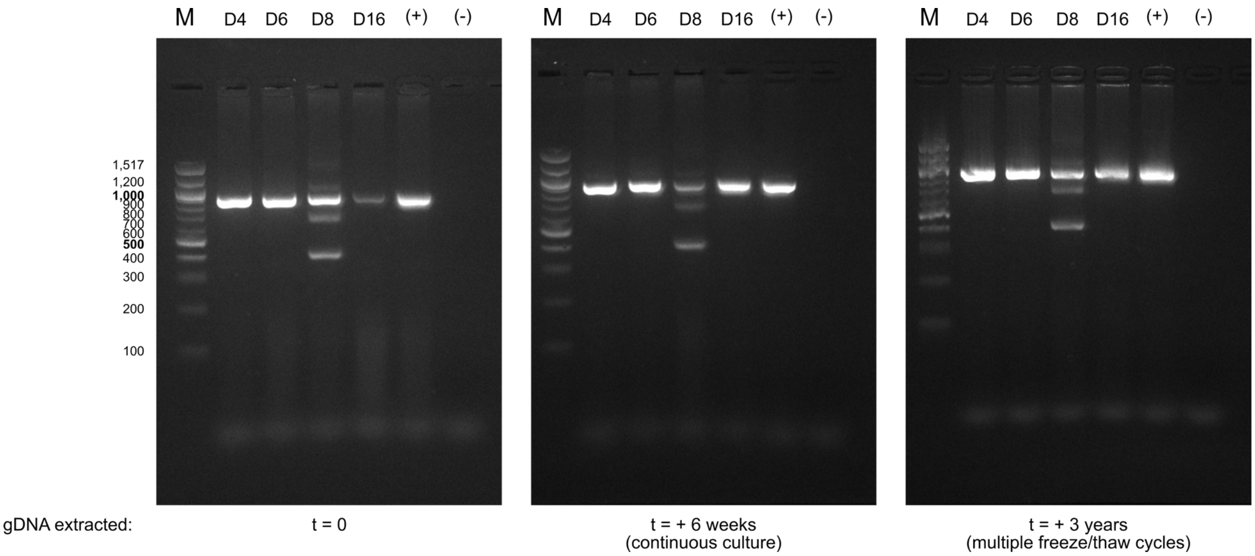

Figure S3: Mutations in the lentiviral integrations encoding MR1-GFP are stable over time. Genomic DNA was extracted from the clonal cell lines at the indicated time points. A region spanning the sgRNA target site was amplified by PCR with a forward primer specific for the tet-inducible construct (5’-TGATAGAGAACGTATGTCGAGT-3’) and a reverse primer binding within the MR1 protein coding region (5’-TTGATGCCCACGCCTG-3’). Amplicons were separated in a 2% agarose gel and imaged with a ChemiDoc MP imaging system (Bio-Rad). Base pair (bp) sizes of the DNA ladder are indicated on the left. Expected band size: ca. 1,000 bp. PCR reaction and gel electrophoresis were performed at the same time for all three sets of gDNA samples. The gDNA shown in the gel on the right was also used for the Illumina sequencing shown in Figures 1D and S4. gDNA from a clonal cell line that expresses the tet-inducible MR1-GFP cassette in the BEAS-2B WT background (i.e. without CRISPR/Cas9 expression) was used as a positive control (+). No DNA template was used as the negative control (-). M = marker.

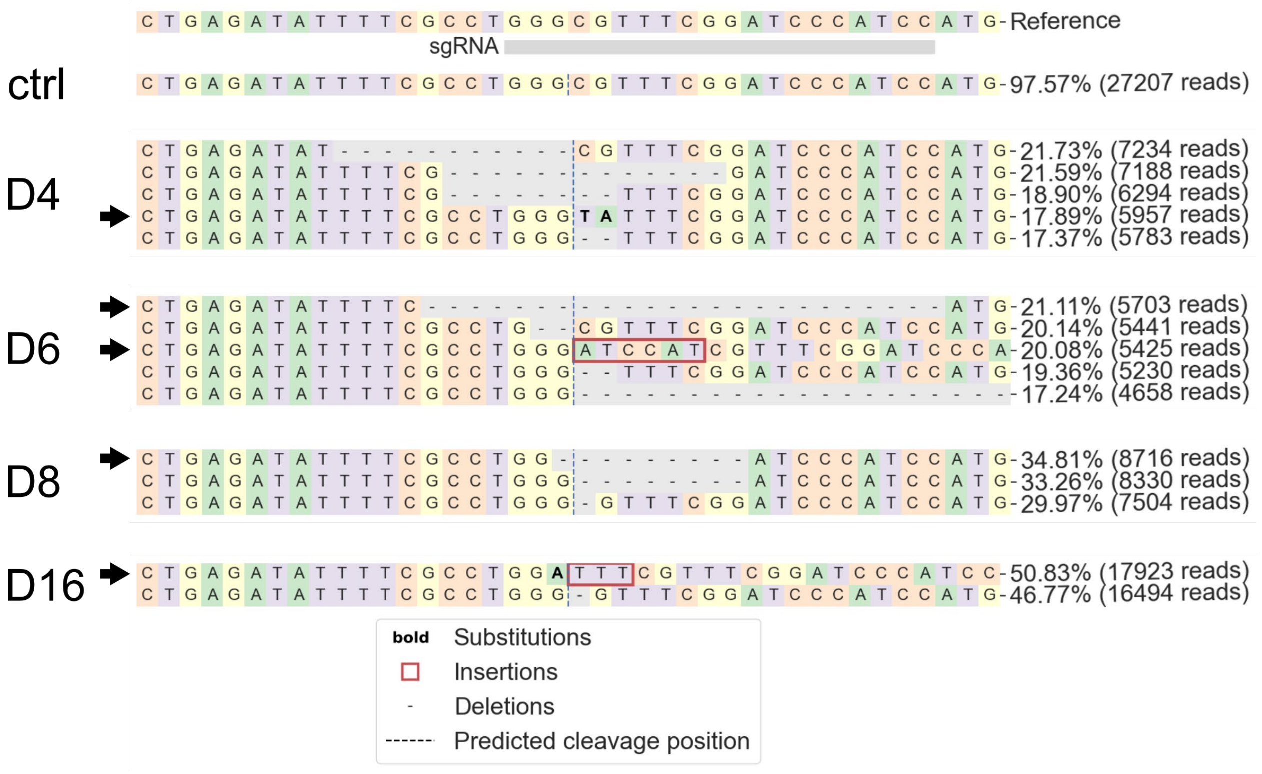

Figure S4: Sequences of all sequenced insertions of the tet MR1-GFP cassette in each clonal cell line. Relates to Figure 1D. The region immediately around the sgRNA target site was amplified by PCR (Fwd: 5'-[Illumina adapter overhang]CACCGGTGGAATTCATGGGGGAA-3'; Rev: 5'-[Illumina adapter overhang] GAGGTTCTCTGCCATCCATGG-3') and 378 bp amplicons were sequenced using the MiSeq System. Data were analyzed and visualized with CRISPResso 2.0 [1]. Contributions of each sequence are shown to the right. Black arrows indicate sequences with an intact reading frame. The same gDNA used here was also used in the righthand gel in Figure S3.

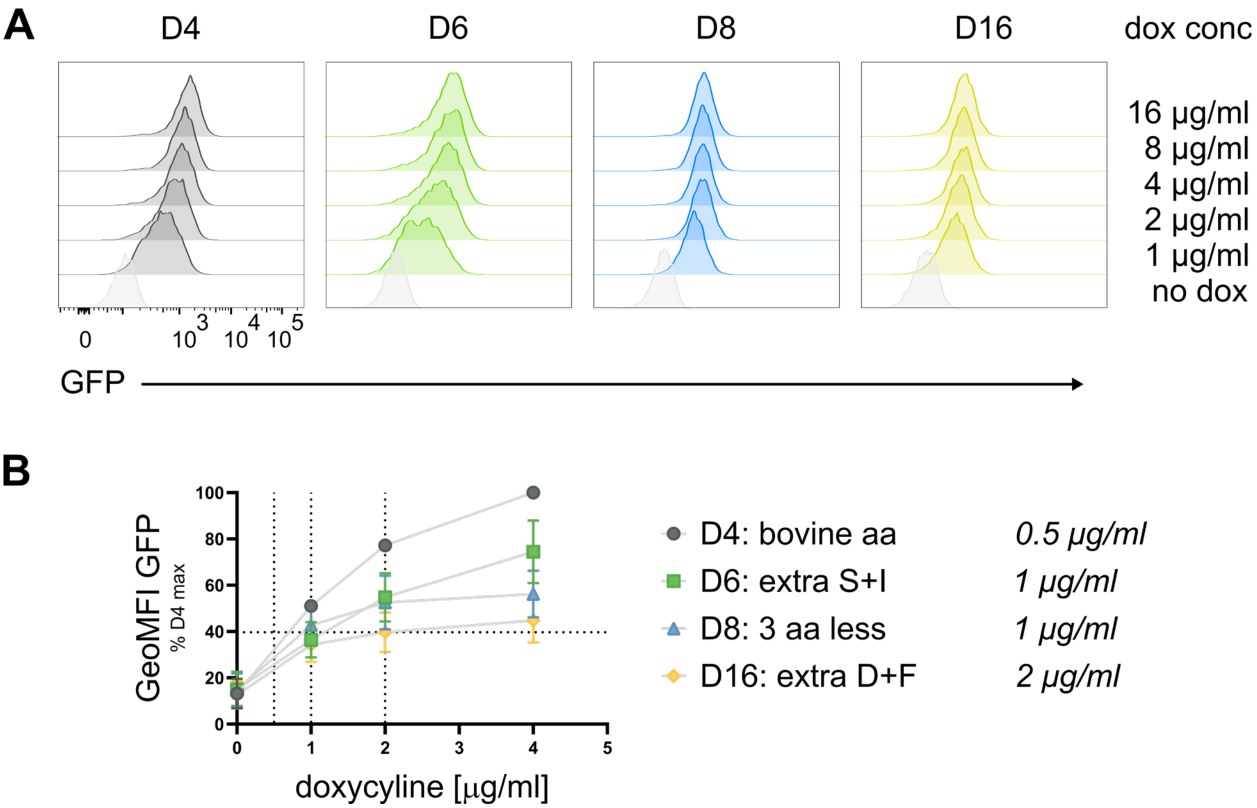

Figure S5: Doxycyline titration to normalize MR1-GFP expression. The four clonal cell lines were incubated with the indicated concentration of doxycycline (dox) overnight and analyzed for GFP expression to determine dox concentrations that induced comparable levels of MR1-GFP. Representative histograms are shown in *A*. Data in *B* are pooled from three independent experiments, normalized to D4 at the highest antigen concentration and shown as mean with SD. Calibration beads were included in each experiment. Based on data in *B*, the following concentrations were routinely used in functional experiments: 0.5 µg/ml for D4, 1 µg/ml for D6 and D8, 2 µg/ml for D16. aa = amino acid.

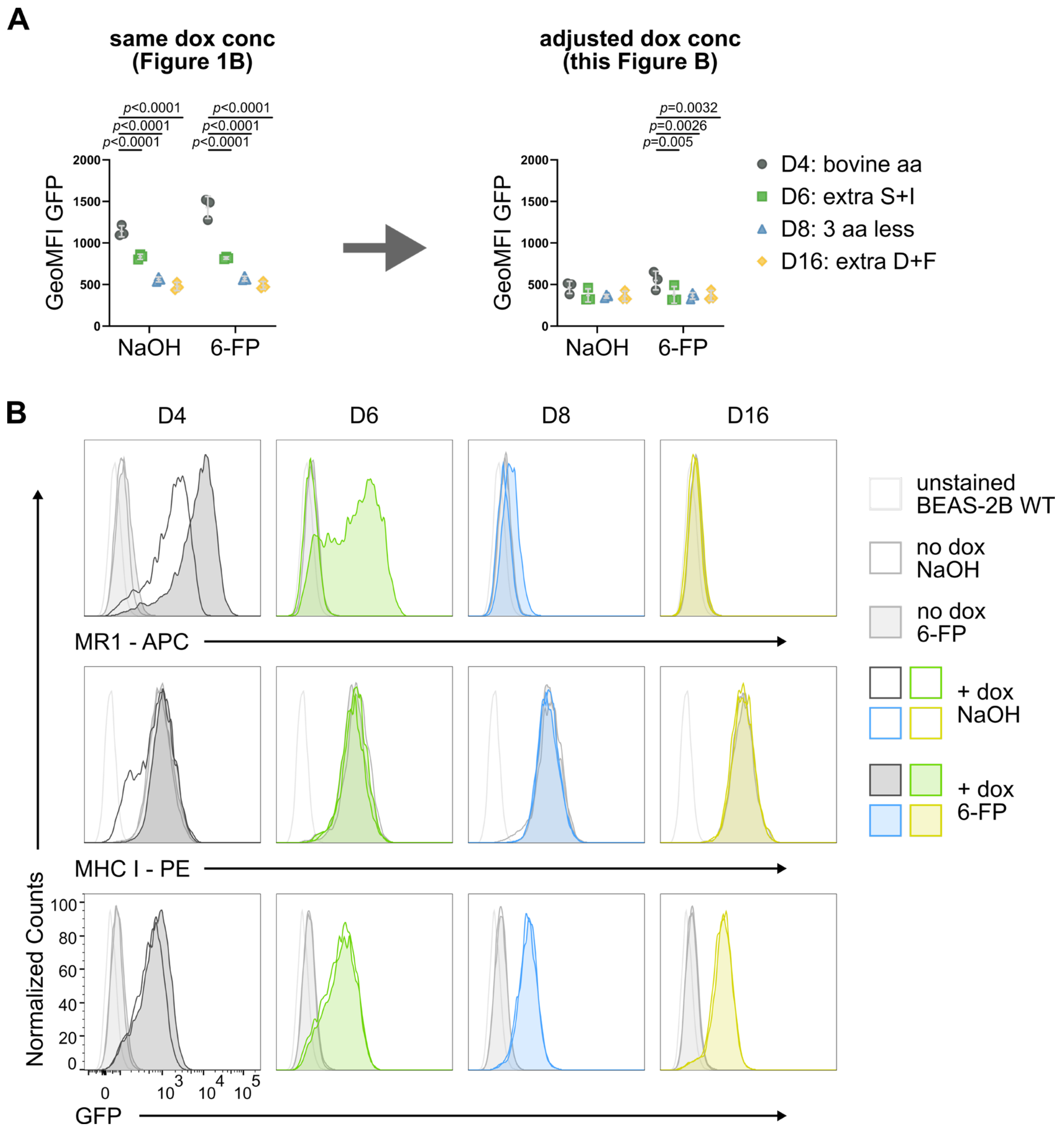

Figure S6: Repeat of flow cytometric analysis with adjusted dox concentrations. Experiment performed as in Figure 1A, but with dox concentrations optimized to normalize MR1-GFP expression (see Figure S5). *A.* Quantification of MR1-GFP geometric mean fluorescence intensity (GeoMFI) pooled from three experiments with 2 µg/ml dox for all four cell lines (including the representative experiment shown in Figure 1A) and three experiments with 0.5, 1, or 2 µg/ml dox (including the representative experiment shown in *B*). Each dot represents a single technical replicate from one independent experiment. Experimental groups were compared by repeated-measures ANOVA with Tukey's multiple comparisons test and statistically significant differences are indicated. Comparisons of all experimental groups are shown in Tables S2 and S3. *B*. Histograms representative of three independent experiments. The same unstained BEAS-2B WT control is shown in each graph for reference. WT = wild type.

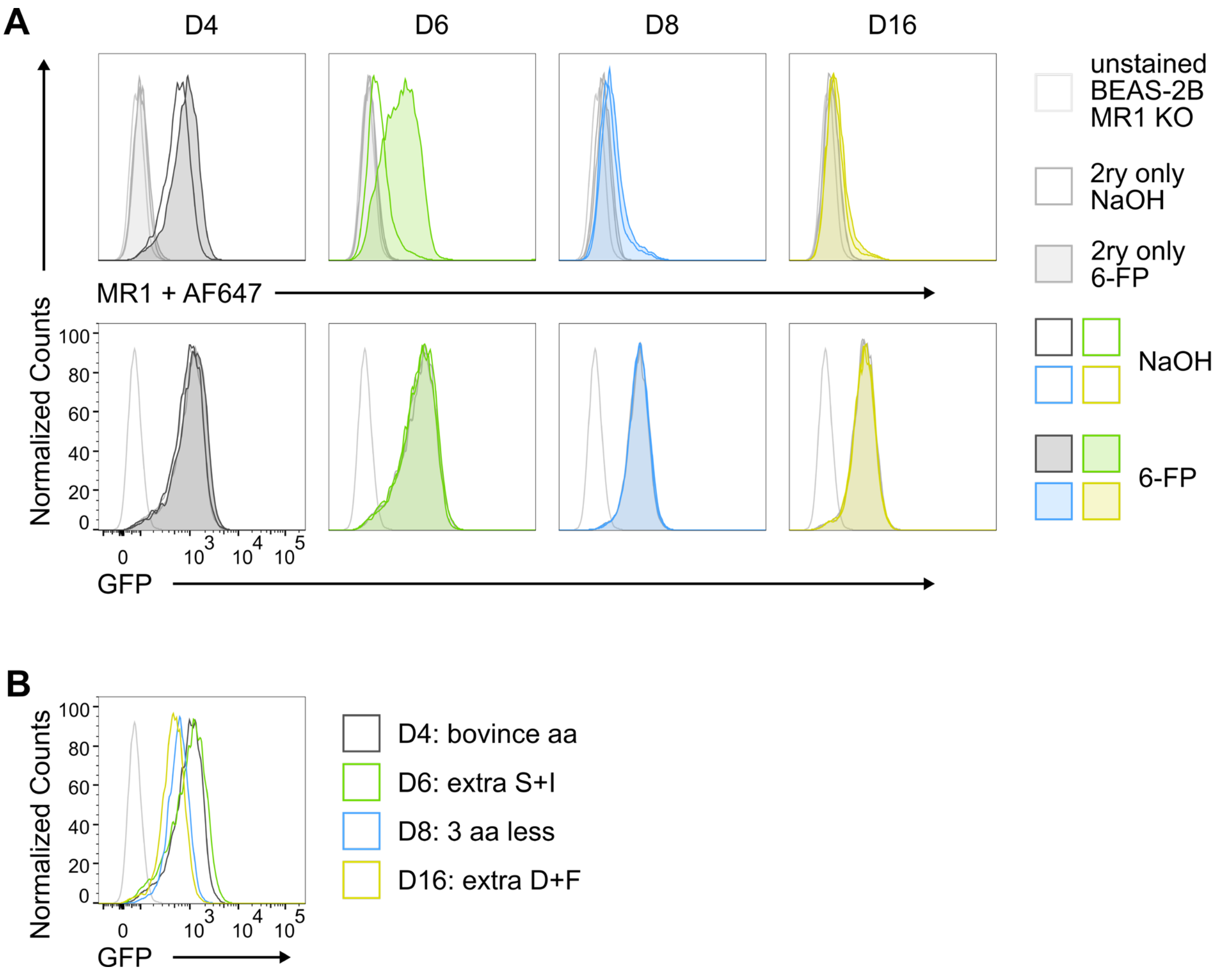

Figure S7: Flow cytometric analysis with a polyclonal anti-MR1 antibody. The four clonal cell lines were induced to express MR1-GFP with twice the adjusted dox concentrations (1 µg/ml, 2 µg/ml, and 4 µg/ml, respectively - see Figure S5 and text) and incubated with 100 µM of 6-FP or solvent control NaOH overnight before staining with a polyclonal anti-MR1 primary antibody followed by AlexaFluor647 (AF647)-coupled secondary antibody (2ry). *A*. Histograms representative of three independent experiments. *B*. Overlay of the GFP histograms of the 2ry only controls + NaOH shown in *A*. The same unstained BEAS-2B MR1 KO control is shown in each graph for reference. KO = knock out; aa = amino acid.

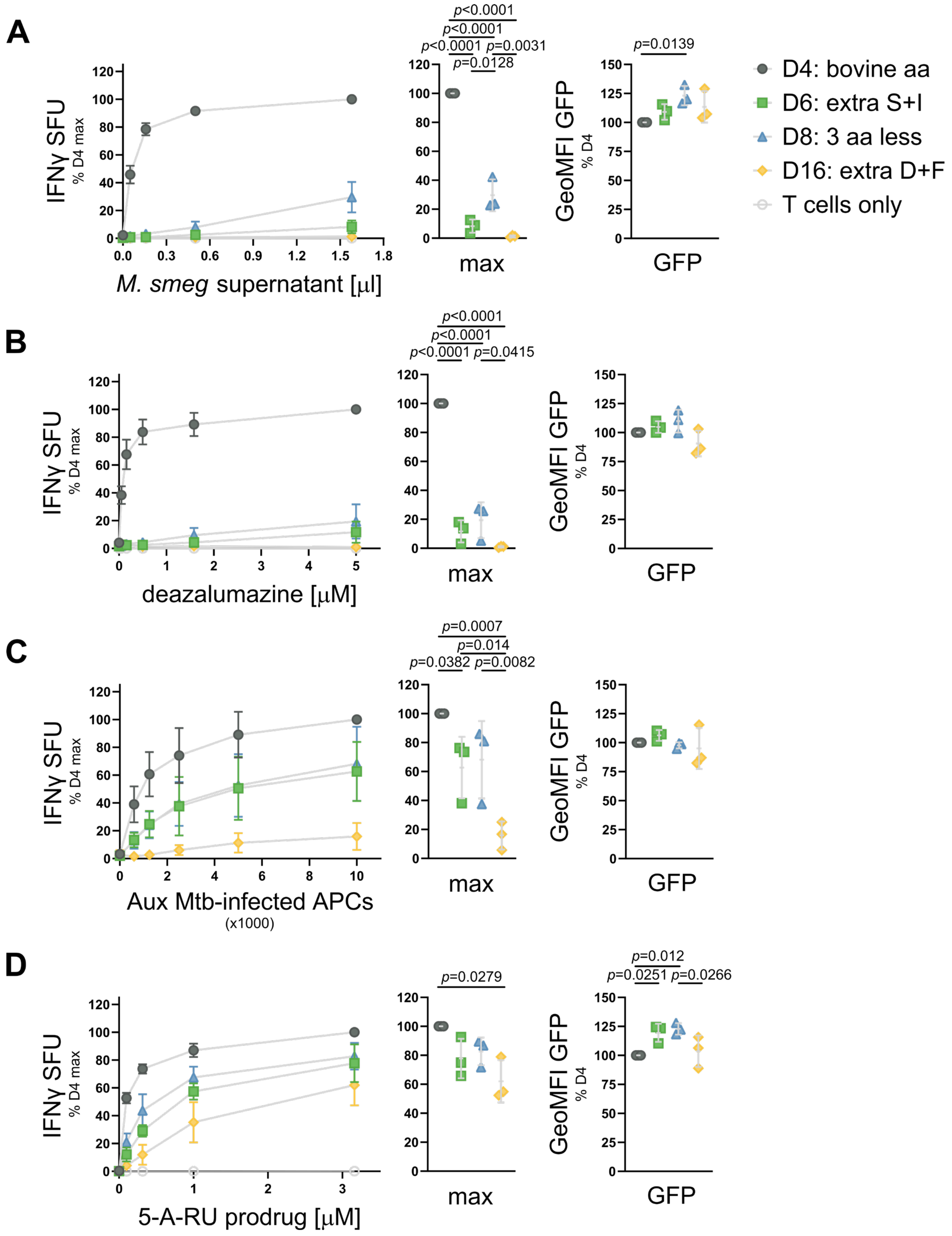
A
B
C
D
IFNγ SFU
% D4 max
M. smeg supernatant [µl]
deazalumazine [µM]
Aux Mtb-infected APCs
(x1000)
5-A-RU prodrug [µM]
max
GFP
GeoMFI GFP
% D4
p<0.0001
p<0.0001
p<0.0001 p=0.0031
p=0.0128
p=0.0139
p<0.0001
p<0.0001
p<0.0001 p=0.0415
p=0.0007
p=0.014
p=0.0382 p=0.0082
p=0.0279
p=0.012
p=0.0251 p=0.0266
D4: bovine aa
D6: extra S+I
D8: 3 aa less
D16: extra D+F
T cells only

Figure S8: Repeat of Figure 2 with MAIT cell clone D426-G11. MR1-GFP expression was induced with adjusted dox concentrations (see Figure S5 and text) overnight and cells were used as APCs in IFNγ ELISPOTs as in Figure 2. Cells were pre-incubated with indicated dilutions of *M.smeg* supernatant (*A*), deazalumazine (*B*), or 5-A-RU prodrug (*D*) for at least one hour before addition of MAIT cell clone D426-G11. APCs in *C* were infected with Aux Mtb overnight and diluted as indicated. MR1-GFP expression at the time of the ELISPOT was measured and is shown to the right of each plot. Each dot represents a single technical replicate from one of three independent experiments. ELISpot data are pooled from three independent experiments, normalized to D4 at the highest antigen concentration and shown as mean with SD. IFNγ responses at the highest antigen concentrations are additionally shown as dot plots where each dot represents the mean of technical duplicates from one independent experiment. Experimental groups were compared by repeated-measures ANOVA with Tukey's multiple comparisons test and statistically significant differences are indicated. Except for *A*, flow cytometry data is the same as shown in Figure 2 as the two MAIT cell clones were assessed in the same experiments using the same APCs. IFN=interferon; SFU=spot forming units; aa=amino acid; GeoMFI = geometric mean fluorescence intensity.

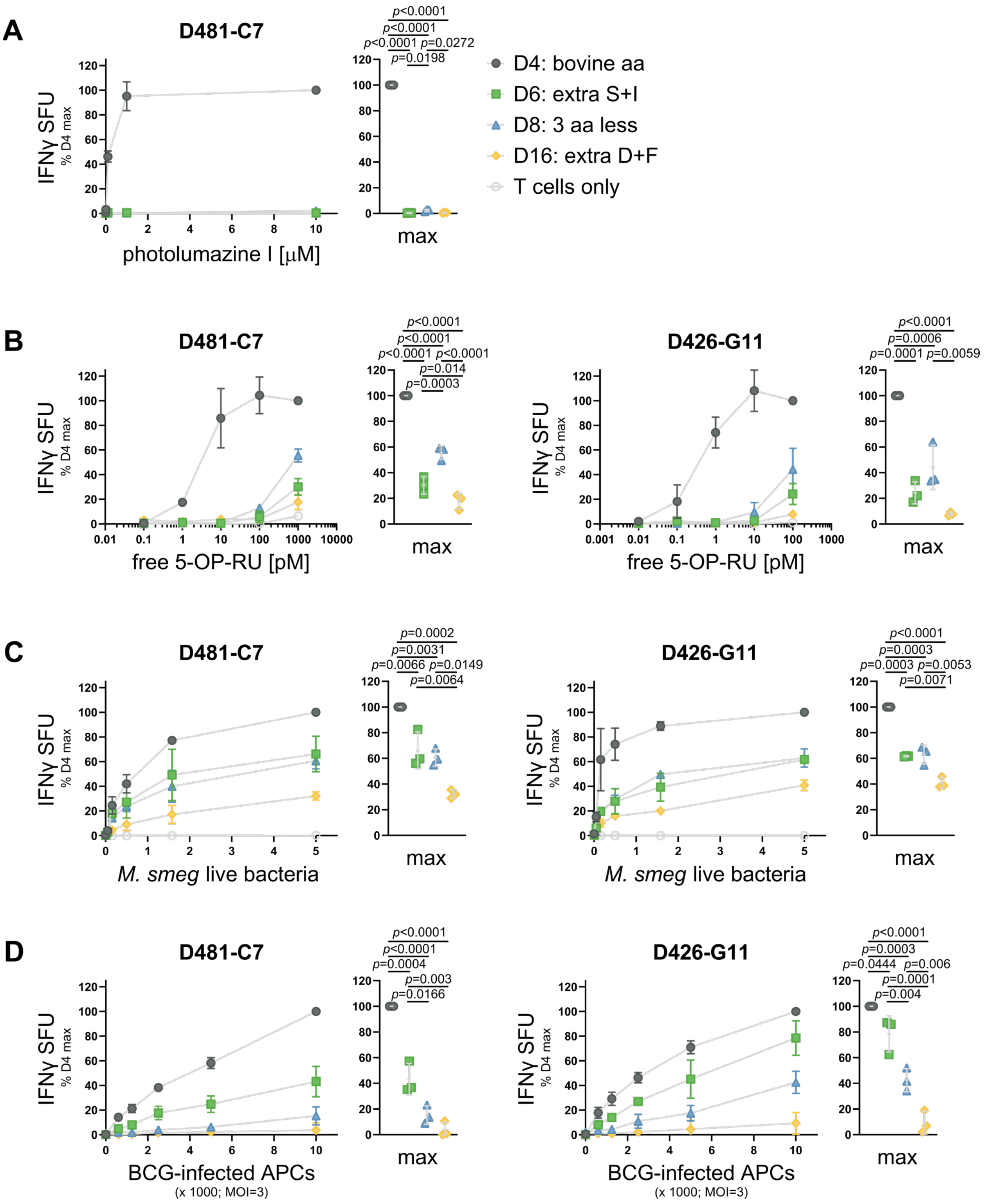
A
D481-C7
IFNγ SFU
% D4 max
photolumazine I [μM]
max
p<0.0001
p<0.0001
p<0.0001 p=0.0272
p=0.0198
D4: bovine aa
D6: extra S+I
D8: 3 aa less
D16: extra D+F
T cells only
B
D481-C7
free 5-OP-RU [pM]
p<0.0001
p<0.0001
p<0.0001 p<0.0001
p=0.014
p=0.0003
D426-G11
p<0.0001
p=0.0006
p=0.0001 p=0.0059
C
D481-C7
M. smeg live bacteria
p=0.0002
p=0.0031
p=0.0066 p=0.0149
p=0.0064
D426-G11
p<0.0001
p=0.0003
p=0.0003 p=0.0053
p=0.0071
D
D481-C7
BCG-infected APCs
(x 1000; MOI=3)
p<0.0001
p<0.0001
p=0.0004
p=0.003
p=0.0166
D426-G11
p<0.0001
p=0.0003
p=0.0444 p=0.006
p=0.0001
p=0.004

Figure S9: Additional antigens tested with clonal cell lines induced with equal concentrations of dox. MR1-GFP expression was induced with 2 µg/ml dox overnight and cells were used as APCs in IFNγ ELISPOTs. Cells were pre-incubated with indicated dilutions of 5-OP-RU (*A*), photolumazine I (*B*), or live *M.smeg* bacteria (*C*) for at least one hour before addition of MAIT cell clones D481-C7 and D426-G11 as indicated. APCs in *D* were infected with BCG at MOI=3 overnight and diluted as indicated. Data are pooled from three independent experiments, normalized to D4 at the highest antigen concentration and shown as mean with SD. IFNγ responses at the highest antigen concentrations are additionally shown as dot plots where each dot represents the mean of technical duplicates from one independent experiment. Experimental groups were compared by repeated-measures ANOVA with Tukey's multiple comparisons test and statistically significant differences are indicated. IFN=interferon; SFU=spot forming units; aa=amino acid.

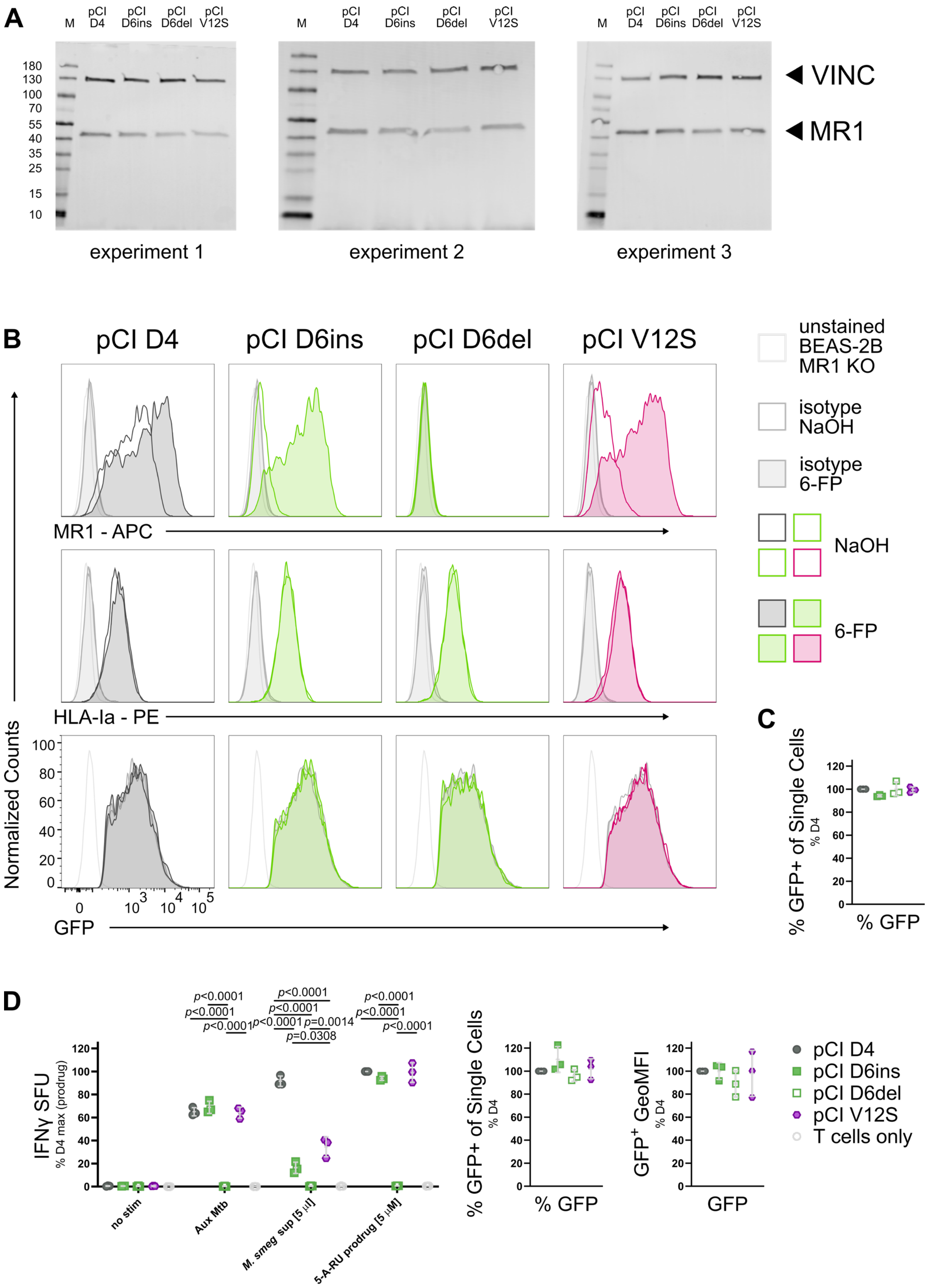
A
M
pCI D4
pCI D6ins
pCI D6del
pCI V12S
180
130
100
70
55
40
35
25
15
10
VINC
MR1
experiment 1
experiment 2
experiment 3
B
pCI D4
pCI D6ins
pCI D6del
pCI V12S
unstained BEAS-2B MR1 KO
isotype NaOH
isotype 6-FP
NaOH
6-FP
MR1 - APC
HLA-Ia - PE
Normalized Counts
GFP
C
% GFP+ of Single Cells
% D4
% GFP
D
IFNγ SFU
% D4 max (prodrug)
p<0.0001
p=0.0014
p=0.0308
no stim
Aux Mtb
M. smeg sup [5 µl]
5-A-RU prodrug [5 µM]
% GFP+ of Single Cells
% GFP
GFP+ GeoMFI
GFP
pCI D4
pCI D6ins
pCI D6del
pCI V12S
T cells only

Figure S10: The D6del mutant does not contribute to the functional phenotype. BEAS-2B MR1 KO cells were transiently transfected with pCI vectors encoding in-frame MR1 mutants identified in the clonal cell lines D4 or D6 (D6ins and D6del) or MR1 V12S with an IRES GFP. Transfected cells were split up and tested for total protein expression by WB (*A*) and MR1 surface expression by flow cytometry (*B*). Transfection efficiency was assessed via the GFP signal (*C*). Data from all three experiments (*A*+*C*) or one representative experiment (*B*) are shown. *D*. BEAS-2B MR1 KO cells were transfected as in *A*-*C* and used as APCs in IFNγ ELISPOTs with the indicated antigens. Data are pooled from three independent experiments, normalized to D4 at the highest antigen concentration and shown as mean with SD. Each dot represents the mean of technical duplicates from one independent experiment. Experimental groups were compared by repeated-measures ANOVA with Tukey's multiple comparisons test for each antigen separately (except for no stim controls), excluding the T cell only condition, and statistically significant differences are indicated. Transfection efficiency and GFP expression levels for cells used in ELISPOTs was assessed by flow cytometry and compared by repeated-measures ANOVA as above. Each dot represents a single technical replicate from one independent experiment. IFN=interferon; SFU=spot forming units; GeoMFI = geometric mean fluorescence intensity.

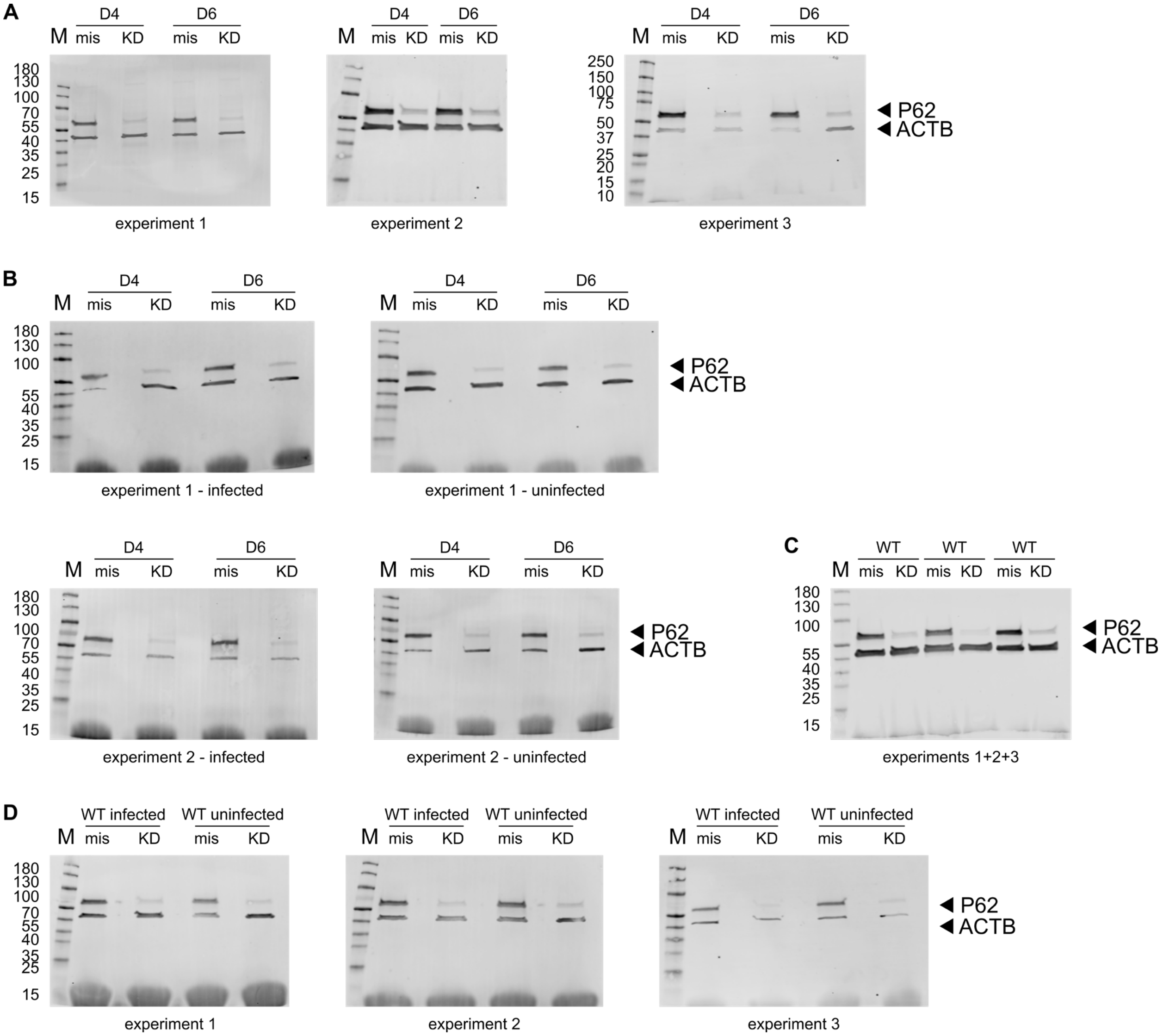

Figure S11: SQSTM1/p62 expression levels in KD experiments. At the time the cells were used in the ELISPOTs shown in Figure 5, some cells were lysed and analyzed by WB to assess SQSTM1/p62 KD on the protein level. Lysates from uninfected cells in *B*+*C* were from approximately 1.1e5 cells each, lysates from infected cells were from approximately 9e4 cells each. Beta actin (ACTB) was used as a loading control. Primary antibodies were from different species and detected in parallel with species-specific secondary antibodies conjugated to distinct IRDyes and both channels exported as greyscale. Molecular weight markers are indicated in kDa on the left. On occasion, lysates from two independent functional experiments were analyzed on the same blot as indicated. Panel labels correspond to panels in Figure 5.

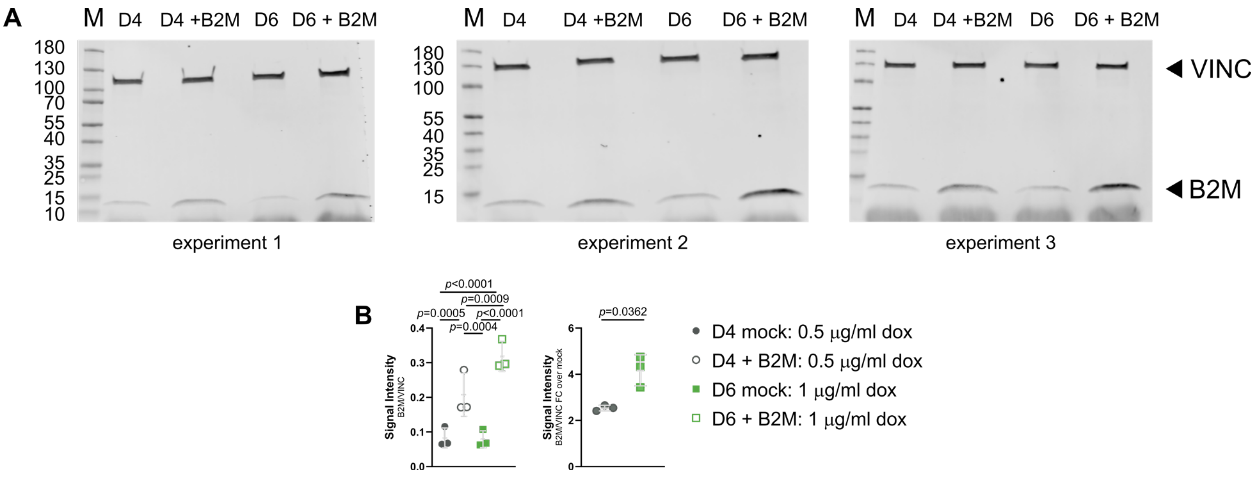

Figure S12: B2M expression levels in OE experiments. At the time the cells were used in the ELISPOTs shown in Figure 6A, some cells were lysed and analyzed by WB to assess B2M overexpression on the protein level. Vinculin (VINC) was used as a loading control. Primary antibodies were from different species and detected in parallel with species-specific secondary antibodies conjugated to distinct IRDyes and both channels exported as greyscale. Molecular weight markers are indicated in kDa on the left. Blots are shown in *A* and signal intensity relative to loading control (*left*) and mock-transfected cells (*right*) is shown in *B*. Each dot represents a single technical replicate from one independent experiment. Western blots for experiments 2 and 3 were performed at the same time. Experimental groups were compared by repeated-measures ANOVA with Tukey's multiple comparisons test (*left*) or two-tailed, paired t-test (*right*) and statistically significant differences are indicated. FC = fold change.

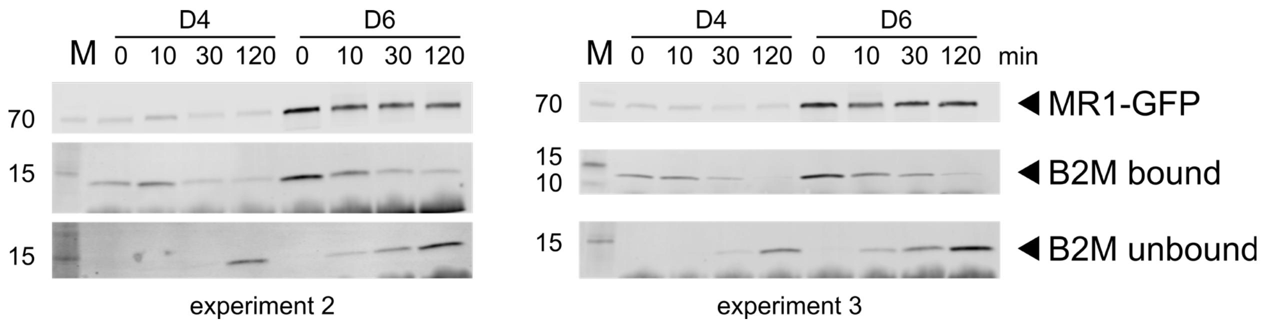

Figure S13: Repeat experiments of the Western blots shown in Figure 6B. WB analysis as in Figure 6B. MR1-GFP was immunoprecipitated from clonal cell lines D4 or D6 induced to express MR1-GFP with 0.5 or 8 µg/ml, respectively, overnight followed by incubation with the same concentrations of dox plus 100 µM 6-FP overnight. Beads were incubated at 37°C for the indicated time periods and MR1 bound to the beads, B2M bound to the beads, and B2M in the supernatant (unbound) were measured by WB. Primary antibodies were detected in parallel with species-specific secondary antibodies conjugated to IRDye800 and both channels exported as greyscale to visualize the molecular weight markers. The 800 channel was exported individually for MR1-GFP. Molecular weight markers are indicated in kDa on the left.

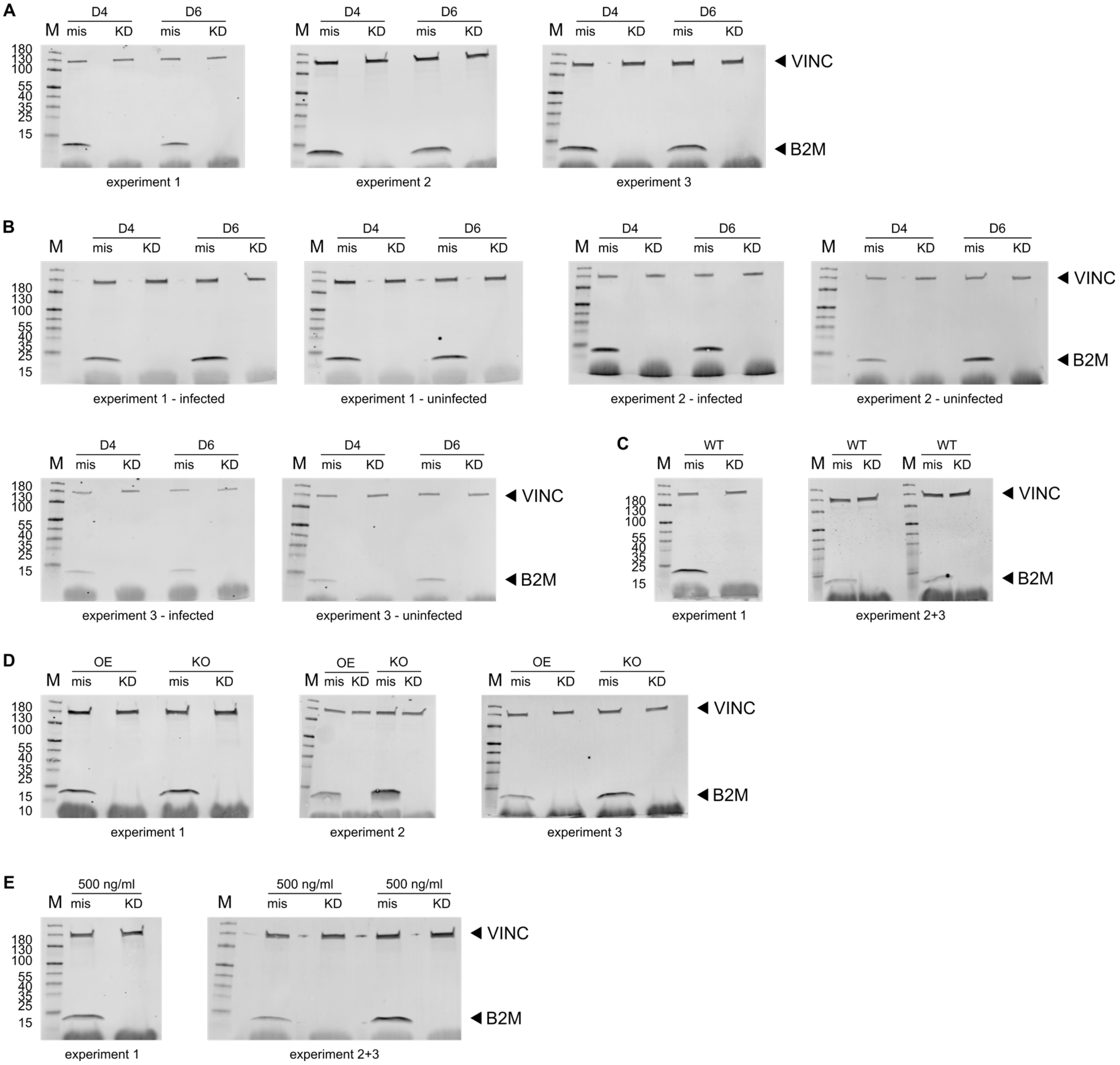

Figure S14: B2M expression levels in KD experiments. At the time the cells were used in the ELISPOTs shown in Figures 7 (*A*+*B*) and 8 (*C*-*E*), some cells were lysed and analyzed by WB to assess B2M KD on the protein level. Vinculin (VINC) was used as a loading control. Primary antibodies were from different species and detected in parallel with species-specific secondary antibodies conjugated to distinct IRDyes and both channels exported as greyscale. Molecular weight markers are indicated in kDa on the left. On occasion, lysates from two independent functional experiments were analyzed on the same blot as indicated.

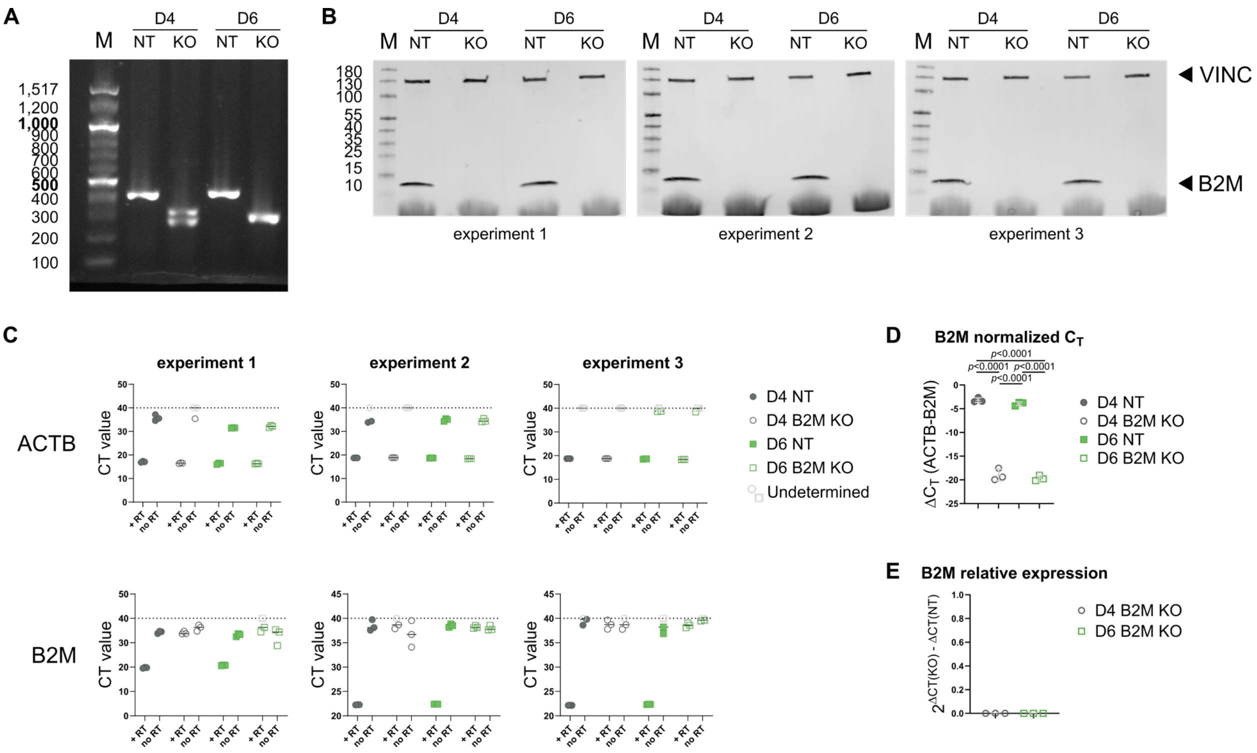

Figure S15: Characterization of B2M CRISPR clones. *A*. Genomic DNA was extracted from the clonal B2M KO cell lines (KO) and their respective controls treated with a non-targeted control RNP (NT) used in Figure 9, and the region targeted by the three sgRNAs was amplified by PCR. The amplicons were run on a 2% agarose gel. Base pair (bp) sizes of the DNA ladder are indicated on the left. Expected band size in WT cells: 451 bp. *B*. B2M protein expression at baseline (no dox treatment) in the four clonal cell lines was analyzed by WB. Primary antibodies against B2M and loading control Vinculin (VINC) were from different species and detected in parallel with species-specific secondary antibodies conjugated to distinct IRDyes and both channels exported as greyscale. Molecular weight markers are indicated in kDa on the left. *C-E*. B2M transcript levels in the four clonal cell lines were measured by qRT-PCR. *C* shows the raw threshold cycle values (C_T) for each sample and its control without reverse transcriptase (noRT) for each experiment. Each dot represents a technical replicate (i.e. one well). “Undetermined” values were set to C_T=40 and colored in light grey for visualization. “Undetermined” values were excluded from the calculations of normalized C_T values (ΔC_T) in *D* and relative expression in *E*. Thus, each dot in *D* and *E* represents the mean of 1-3 technical replicates from one of the experiments shown in *C*. Experimental groups in *D* were compared by repeated-measures ANOVA with Tukey’s multiple comparisons test and statistically significant differences are indicated.

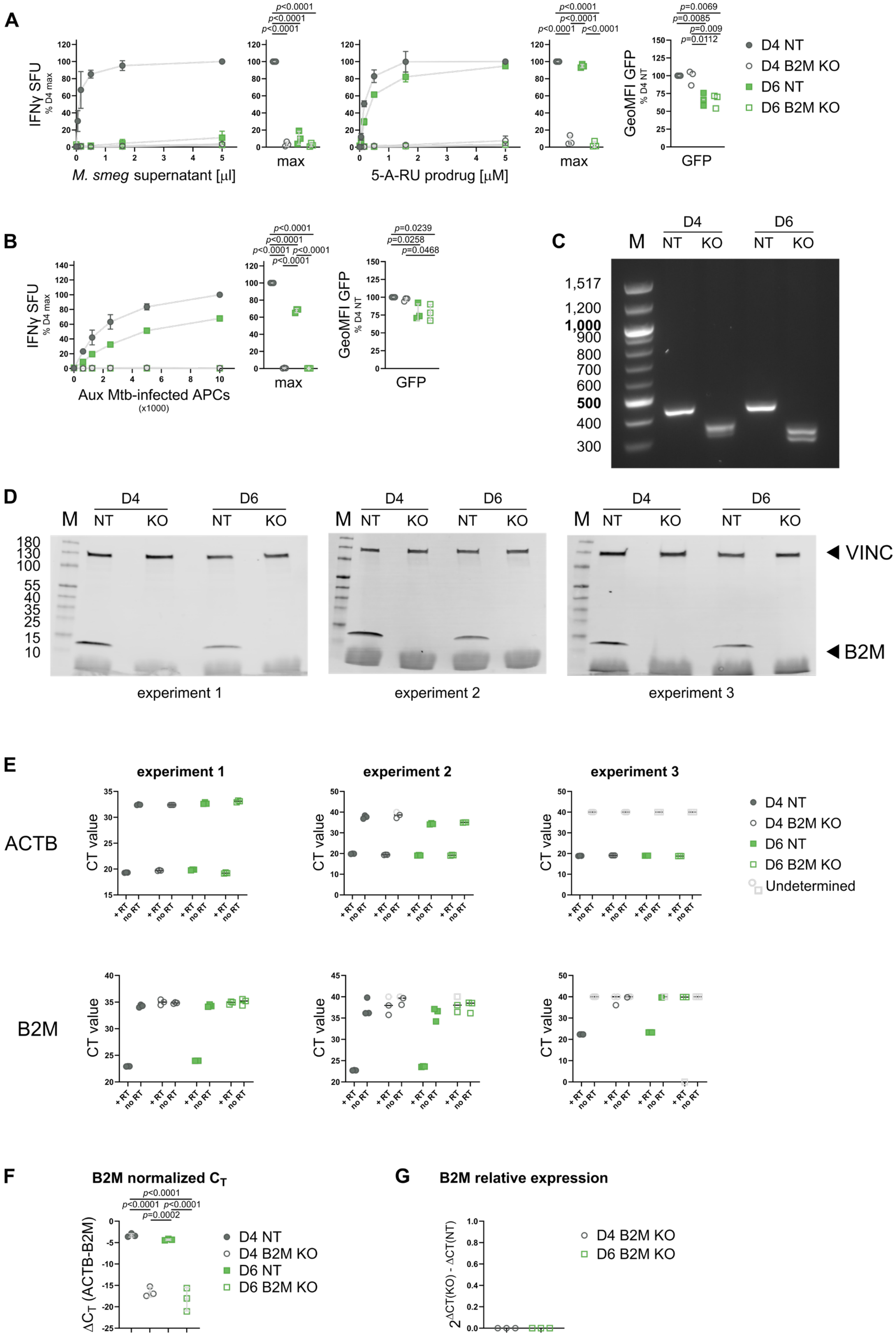
A
IFNγ SFU
% D4 max
M. smeg supernatant [µl]
max
5-A-RU prodrug [µM]
GeoMFI GFP
% D4 NT
GFP
p<0.0001
p=0.0069
p=0.0085
p=0.009
p=0.0112
D4 NT
D4 B2M KO
D6 NT
D6 B2M KO
B
Aux Mtb-infected APCs
(x1000)
p=0.0239
p=0.0258
p=0.0468
C
M
D4
D6
NT
KO
1,517
1,200
1,000
900
800
700
600
500
400
300
D
180
130
100
55
40
35
25
15
10
VINC
B2M
experiment 1
experiment 2
experiment 3
E
ACTB
B2M
CT value
+ RT
no RT
Undetermined
F
B2M normalized C_T
p=0.0002
ΔC_T (ACTB-B2M)
G
B2M relative expression
$2^{\Delta CT(KO) - \Delta CT(NT)}$

Figure S16: Analysis of a second set of B2M KO clonal cell lines. To confirm the B2M KO phenotype, a second set of B2M KO (KO) and non-target control (NT) clonal cell lines derived from D4 and D6 were analyzed as in Figures 9 and S15. *A*. Clonal cell lines were induced to express MR1-GFP with adjusted dox concentrations (see Figure S5) and used as APCs in IFNγ ELISPOTs with the indicated antigens. MR1-GFP expression was measured by flow cytometry at the time of the ELISPOT and shown to the right. Each dot represents a single technical replicate from one of three independent experiments. ELISpot data are pooled from three independent experiments, normalized to D4 at the highest antigen concentration and shown as mean with SD. IFNγ responses at the highest antigen concentrations are additionally shown as dot plots where each dot represents the mean of technical duplicates from one independent experiment. *C*. Genomic DNA was extracted and the region targeted by the three sgRNAs was amplified by PCR. The amplicons were run on a 2% agarose gel. Base pair (bp) sizes of the DNA ladder are indicated on the left. Expected band size in WT cells: 451 bp. *D*. B2M protein expression at baseline (no dox treatment) in the four clonal cell lines was analyzed by WB. Primary antibodies against B2M and loading control Vinculin (VINC) were from different species and detected in parallel with species-specific secondary antibodies conjugated to distinct IRDyes and both channels exported as greyscale. Molecular weight markers are indicated in kDa on the left. *E-G*. B2M transcript levels in the four clonal cell lines were measured by qRT-PCR. *E* shows the raw threshold cycle values (C_T) for each sample and its control without reverse transcriptase (noRT) for each experiment. Each dot represents a technical replicate (i.e. one well). “Undetermined” values were set to C_T=40 and colored in light grey for visualization. “Undetermined” values were excluded from the calculations of normalized C_T values (ΔC_T) in *F* and relative expression in *G*. Thus, each dot in *F* and *G* represents the mean of 1-3 technical replicates from one of the experiments shown in *E*. Experimental groups in *A, B* + *F* were compared by repeated-measures ANOVA with Tukey’s multiple comparisons test and statistically significant differences are indicated. IFN=interferon; SFU=spot forming units; GeoMFI = geometric mean fluorescence intensity.

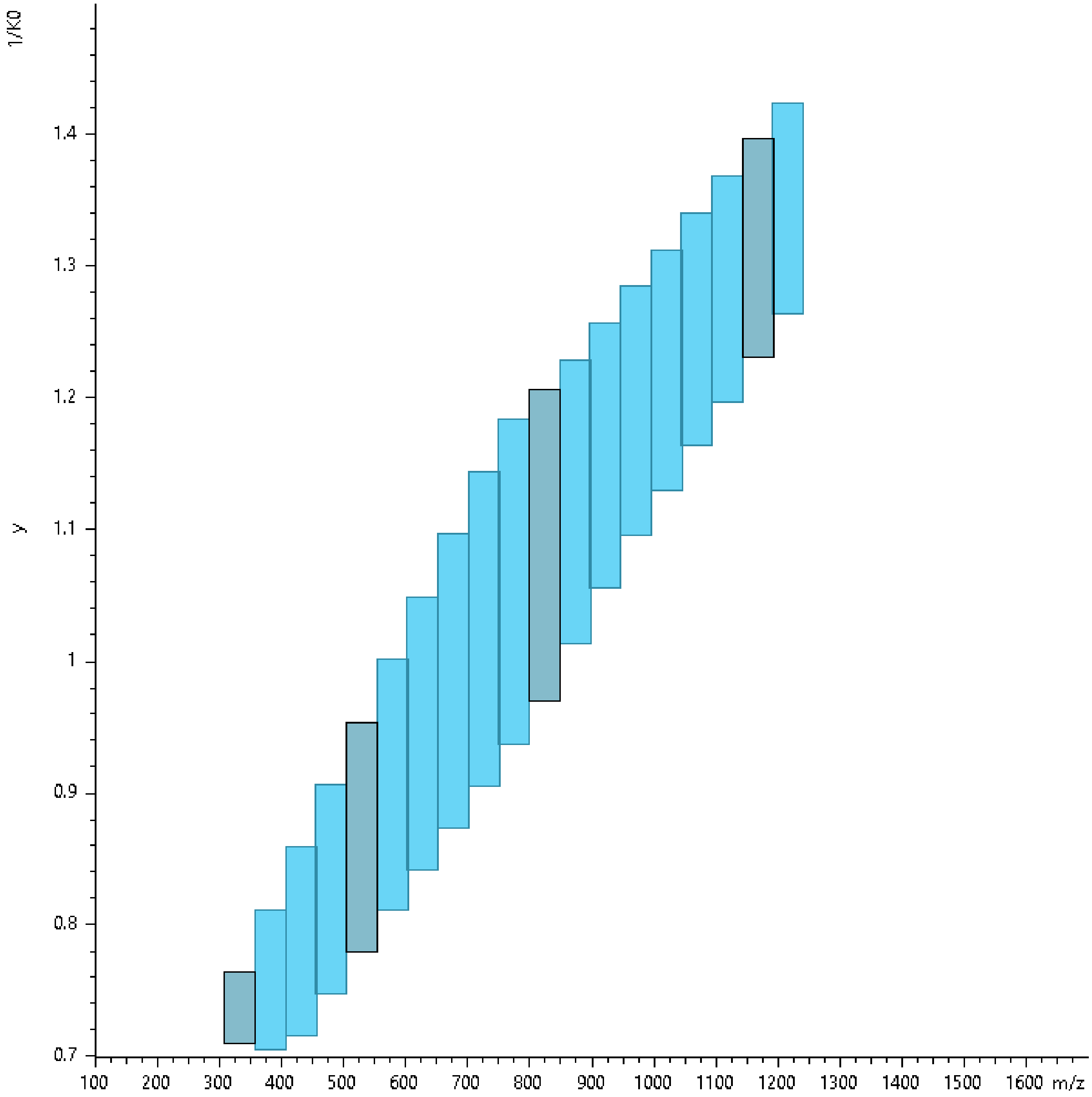

Figure S17: dia-PASEF acquisition schemes on Bruker timsTOF Flex. Scheme visualization was performed using the Bruker timsControl software.

Table S1: Nonlinear fit Table of results for Fig 1B. Table of Results for nonlinear regression analysis to determine whether data sets differ from each other. Straight line models were fit using least squares regression and compared using the extra sum-of-squares F test in Graphpad Prism 10.4.1.

**Comparison of Fits**					
Null hypothesis	One curve for all data sets				
Alternative hypothesis	Different curve for at least one data set				
P value	0.0126				
F (DFn, DFd)	2.953 (6, 70)				
**Different curve for at least one data set**					
Best-fit values	D4	D6	D8	D16	Global
YIntercept	98.76	98.34	97.63	96.94	
Slope	-2.941	-2.848	-2.44	-2.692	
95% CI (profile likelihood)					
YIntercept	92.00 to 105.5	92.60 to 104.1	92.80 to 102.5	91.94 to 101.9	
Slope	-3.348 to -2.535	-3.198 to -2.498	-2.732 to -2.149	-2.994 to -2.390	
Goodness of Fit					
Degrees of Freedom	17	17	18	18	
R squared	0.932	0.9454	0.9451	0.9512	
Sum of Squares	854.4	620	469.7	504.1	
Sy.x	7.089	6.039	5.108	5.292	
**One curve for all data sets**					
Best-fit values					
YIntercept	97.94	97.94	97.94	97.94	97.94
Slope	-2.728	-2.728	-2.728	-2.728	-2.728
95% CI (profile likelihood)					
YIntercept	95.08 to 100.8	95.08 to 100.8	95.08 to 100.8	95.08 to 100.8	95.08 to 100.8
Slope	-2.900 to -2.555	-2.900 to -2.555	-2.900 to -2.555	-2.900 to -2.555	-2.900 to -2.555
Goodness of Fit					
Degrees of Freedom					76
R squared	0.9195	0.9409	0.8976	0.9506	0.929
Sum of Squares	1011	671	875.3	510.4	3068
Sy.x					6.354
Constraints					
YIntercept	YIntercept is shared	YIntercept is shared	YIntercept is shared	YIntercept is shared	
Slope	Slope is shared	Slope is shared	Slope is shared	Slope is shared	
Number of points					
# of X values	20	20	20	20	
# Y values analyzed	19	19	20	20	

Table S2: ANOVA table for GFP GeoMFI with equal dox concentrations. Relates to Fig 1A and Fig S6A. Table of Results for repeated-measures one-way ANOVA with Tukey's multiple comparisons test comparing GFP GeoMFI between experimental groups in Graphpad Prism 10.4.1.

**Repeated measures ANOVA summary**					
Assume sphericity?	Yes				
F	138				
P value	<0.0001				
P value summary	****				
Statistically significant (P < 0.05)?	Yes				
R squared	0.9857				
Was the matching effective?					
F	4.558				
P value	0.0299				
P value summary	*				
Is there significant matching (P < 0.05)?	Yes				
R squared	0.009219				
ANOVA table	SS	DF	MS	F (DFn, DFd)	P value
Treatment (between columns)	2459701	7	351386	F (7, 14) = 138.0	P<0.0001
Individual (between rows)	23219	2	11610	F (2, 14) = 4.558	P=0.0299
Residual (random)	35660	14	2547		
Total	2518579	23			
Data summary					
Number of treatments (columns)	8				
Number of subjects (rows)	3				
Number of missing values	0				

Number of families			1				
Number of comparisons per family			28				
Alpha			0.05				
**Tukey's multiple comparisons test**							
	Mean Diff.	95.00% CI of diff.	Below threshold?	Summary	Adjusted P Value		
D4 NaOH vs. D6 NaOH	306.3	160.9 to 451.7	Yes	****	<0.0001		
D4 NaOH vs. D8 NaOH	580	434.6 to 725.4	Yes	****	<0.0001		
D4 NaOH vs. D16 NaOH	663.3	517.9 to 808.7	Yes	****	<0.0001		
D4 NaOH vs. D4 6FP	-286.7	-432.1 to -141.3	Yes	***	0.0001		
D4 NaOH vs. D6 6FP	321.7	176.3 to 467.1	Yes	****	<0.0001		
D4 NaOH vs. D8 6FP	568.3	422.9 to 713.7	Yes	****	<0.0001		
D4 NaOH vs. D16 6FP	651	505.6 to 796.4	Yes	****	<0.0001		

D6 NaOH vs. D8 NaOH	273.7	128.3 to 419.1	Yes	***	0.0002			
D6 NaOH vs. D16 NaOH	357	211.6 to 502.4	Yes	****	<0.0001			
D6 NaOH vs. D4 6FP	-593	-738.4 to -447.6	Yes	****	<0.0001			
D6 NaOH vs. D6 6FP	15.33	-130.1 to 160.7	No	ns	>0.9999			
D6 NaOH vs. D8 6FP	262	116.6 to 407.4	Yes	***	0.0004			
D6 NaOH vs. D16 6FP	344.7	199.3 to 490.1	Yes	****	<0.0001			
D8 NaOH vs. D16 NaOH	83.33	-62.08 to 228.7	No	ns	0.5016			
D8 NaOH vs. D4 6FP	-866.7	-1012 to -721.3	Yes	****	<0.0001			
D8 NaOH vs. D6 6FP	-258.3	-403.7 to -112.9	Yes	***	0.0004			
D8 NaOH vs. D8 6FP	-11.67	-157.1 to 133.7	No	ns	>0.9999			
D8 NaOH vs. D16 6FP	71	-74.41 to 216.4	No	ns	0.6745			
D16 NaOH vs. D4 6FP	-950	-1095 to -804.6	Yes	****	<0.0001			
D16 NaOH vs. D6 6FP	-341.7	-487.1 to -196.3	Yes	****	<0.0001			
D16 NaOH vs. D8 6FP	-95	-240.4 to 50.41	No	ns	0.3542			
D16 NaOH vs. D16 6FP	-12.33	-157.7 to 133.1	No	ns	>0.9999			
D4 6FP vs. D6 6FP	608.3	462.9 to 753.7	Yes	****	<0.0001			
D4 6FP vs. D8 6FP	855	709.6 to 1000	Yes	****	<0.0001			
D4 6FP vs. D16 6FP	937.7	792.3 to 1083	Yes	****	<0.0001			
D6 6FP vs. D8 6FP	246.7	101.3 to 392.1	Yes	***	0.0007			
D6 6FP vs. D16 6FP	329.3	183.9 to 474.7	Yes	****	<0.0001			
D8 6FP vs. D16 6FP	82.67	-62.74 to 228.1	No	ns	0.5107			
**Test details**	Mean 1	Mean 2	Mean Diff.	SE of diff.	n1	n2	q	DF
D4 NaOH vs. D6 NaOH	1141	834.3	306.3	41.21	3	3	10.51	14
D4 NaOH vs. D8 NaOH	1141	560.7	580	41.21	3	3	19.91	14
D4 NaOH vs. D16 NaOH	1141	477.3	663.3	41.21	3	3	22.77	14
D4 NaOH vs. D4 6FP	1141	1427	-286.7	41.21	3	3	9.838	14
D4 NaOH vs. D6 6FP	1141	819	321.7	41.21	3	3	11.04	14
D4 NaOH vs. D8 6FP	1141	572.3	568.3	41.21	3	3	19.5	14
D4 NaOH vs. D16 6FP	1141	489.7	651	41.21	3	3	22.34	14
D6 NaOH vs. D8 NaOH	834.3	560.7	273.7	41.21	3	3	9.392	14
D6 NaOH vs. D16 NaOH	834.3	477.3	357	41.21	3	3	12.25	14
D6 NaOH vs. D4 6FP	834.3	1427	-593	41.21	3	3	20.35	14
D6 NaOH vs. D6 6FP	834.3	819	15.33	41.21	3	3	0.5262	14
D6 NaOH vs. D8 6FP	834.3	572.3	262	41.21	3	3	8.992	14
D6 NaOH vs. D16 6FP	834.3	489.7	344.7	41.21	3	3	11.83	14
D8 NaOH vs. D16 NaOH	560.7	477.3	83.33	41.21	3	3	2.86	14
D8 NaOH vs. D4 6FP	560.7	1427	-866.7	41.21	3	3	29.74	14
D8 NaOH vs. D6 6FP	560.7	819	-258.3	41.21	3	3	8.866	14
D8 NaOH vs. D8 6FP	560.7	572.3	-11.67	41.21	3	3	0.4004	14
D8 NaOH vs. D16 6FP	560.7	489.7	71	41.21	3	3	2.437	14

D16 NaOH vs. D4 6FP	477.3	1427	-950	41.21	3	3	32.6	14
D16 NaOH vs. D6 6FP	477.3	819	-341.7	41.21	3	3	11.73	14
D16 NaOH vs. D8 6FP	477.3	572.3	-95	41.21	3	3	3.26	14
D16 NaOH vs. D16 6FP	477.3	489.7	-12.33	41.21	3	3	0.4233	14
D4 6FP vs. D6 6FP	1427	819	608.3	41.21	3	3	20.88	14
D4 6FP vs. D8 6FP	1427	572.3	855	41.21	3	3	29.34	14
D4 6FP vs. D16 6FP	1427	489.7	937.7	41.21	3	3	32.18	14
D6 6FP vs. D8 6FP	819	572.3	246.7	41.21	3	3	8.465	14
D6 6FP vs. D16 6FP	819	489.7	329.3	41.21	3	3	11.3	14
D8 6FP vs. D16 6FP	572.3	489.7	82.67	41.21	3	3	2.837	14

Table S3: ANOVA table for GFP GeoMFI with adjusted dox concentrations. Relates to Fig S6. Table of Results for repeated-measures one-way ANOVA with Tukey's multiple comparisons test comparing GFP GeoMFI between experimental groups in Graphpad Prism 10.4.1.

**Repeated measures ANOVA summary**					
Assume sphericity?	Yes				
F	7.531				
P value	0.0007				
P value summary	***				
Statistically significant (P < 0.05)?	Yes				
R squared	0.7902				
Was the matching effective?					
F	15.94				
P value	0.0002				
P value summary	***				
Is there significant matching (P < 0.05)?	Yes				
R squared	0.3233				
ANOVA table	SS	DF	MS	F (DFn, DFd)	P value
Treatment (between columns)	100910	7	14416	F (7, 14) = 7.531	P=0.0007
Individual (between rows)	61021	2	30511	F (2, 14) = 15.94	P=0.0002
Residual (random)	26798	14	1914		
Total	188729	23			
Data summary					
Number of treatments (columns)	8				
Number of subjects (rows)	3				
Number of missing values	0				

Number of families			1				
Number of comparisons per family			28				
Alpha			0.05				
**Tukey's multiple comparisons test**							
	Mean Diff.	95.00% CI of diff.	Below threshold?	Summary	Adjusted P Value		
D4 NaOH vs. D6 NaOH	97.33	-28.72 to 223.4	No	ns	0.1929		
D4 NaOH vs. D8 NaOH	107.3	-18.72 to 233.4	No	ns	0.123		
D4 NaOH vs. D16 NaOH	106	-20.05 to 232.1	No	ns	0.1308		
D4 NaOH vs. D4 6FP	-82.33	-208.4 to 43.72	No	ns	0.3544		
D4 NaOH vs. D6 6FP	89.67	-36.39 to 215.7	No	ns	0.2663		

D4 NaOH vs. D8 6FP	103	-23.05 to 229.1	No	ns	0.15			
D4 NaOH vs. D16 6FP	98.67	-27.39 to 224.7	No	ns	0.1819			
D6 NaOH vs. D8 NaOH	10	-116.1 to 136.1	No	ns	>0.9999			
D6 NaOH vs. D16 NaOH	8.667	-117.4 to 134.7	No	ns	>0.9999			
D6 NaOH vs. D4 6FP	-179.7	-305.7 to -53.61	Yes	**	0.0034			
D6 NaOH vs. D6 6FP	-7.667	-133.7 to 118.4	No	ns	>0.9999			
D6 NaOH vs. D8 6FP	5.667	-120.4 to 131.7	No	ns	>0.9999			
D6 NaOH vs. D16 6FP	1.333	-124.7 to 127.4	No	ns	>0.9999			
D8 NaOH vs. D16 NaOH	-1.333	-127.4 to 124.7	No	ns	>0.9999			
D8 NaOH vs. D4 6FP	-189.7	-315.7 to -63.61	Yes	**	0.0021			
D8 NaOH vs. D6 6FP	-17.67	-143.7 to 108.4	No	ns	0.9995			
D8 NaOH vs. D8 6FP	-4.333	-130.4 to 121.7	No	ns	>0.9999			
D8 NaOH vs. D16 6FP	-8.667	-134.7 to 117.4	No	ns	>0.9999			
D16 NaOH vs. D4 6FP	-188.3	-314.4 to -62.28	Yes	**	0.0022			
D16 NaOH vs. D6 6FP	-16.33	-142.4 to 109.7	No	ns	0.9997			
D16 NaOH vs. D8 6FP	-3	-129.1 to 123.1	No	ns	>0.9999			
D16 NaOH vs. D16 6FP	-7.333	-133.4 to 118.7	No	ns	>0.9999			
D4 6FP vs. D6 6FP	172	45.95 to 298.1	Yes	**	0.005			
D4 6FP vs. D8 6FP	185.3	59.28 to 311.4	Yes	**	0.0026			
D4 6FP vs. D16 6FP	181	54.95 to 307.1	Yes	**	0.0032			
D6 6FP vs. D8 6FP	13.33	-112.7 to 139.4	No	ns	>0.9999			
D6 6FP vs. D16 6FP	9	-117.1 to 135.1	No	ns	>0.9999			
D8 6FP vs. D16 6FP	-4.333	-130.4 to 121.7	No	ns	>0.9999			
**Test details**	Mean 1	Mean 2	Mean Diff.	SE of diff.	n1	n2	q	DF
D4 NaOH vs. D6 NaOH	466	368.7	97.33	35.72	3	3	3.853	14
D4 NaOH vs. D8 NaOH	466	358.7	107.3	35.72	3	3	4.249	14
D4 NaOH vs. D16 NaOH	466	360	106	35.72	3	3	4.196	14
D4 NaOH vs. D4 6FP	466	548.3	-82.33	35.72	3	3	3.259	14
D4 NaOH vs. D6 6FP	466	376.3	89.67	35.72	3	3	3.55	14
D4 NaOH vs. D8 6FP	466	363	103	35.72	3	3	4.078	14
D4 NaOH vs. D16 6FP	466	367.3	98.67	35.72	3	3	3.906	14
D6 NaOH vs. D8 NaOH	368.7	358.7	10	35.72	3	3	0.3959	14
D6 NaOH vs. D16 NaOH	368.7	360	8.667	35.72	3	3	0.3431	14
D6 NaOH vs. D4 6FP	368.7	548.3	-179.7	35.72	3	3	7.113	14
D6 NaOH vs. D6 6FP	368.7	376.3	-7.667	35.72	3	3	0.3035	14
D6 NaOH vs. D8 6FP	368.7	363	5.667	35.72	3	3	0.2243	14
D6 NaOH vs. D16 6FP	368.7	367.3	1.333	35.72	3	3	0.05278	14
D8 NaOH vs. D16 NaOH	358.7	360	-1.333	35.72	3	3	0.05278	14

D8 NaOH vs. D4 6FP	358.7	548.3	-189.7	35.72	3	3	7.509	14
D8 NaOH vs. D6 6FP	358.7	376.3	-17.67	35.72	3	3	0.6994	14
D8 NaOH vs. D8 6FP	358.7	363	-4.333	35.72	3	3	0.1716	14
D8 NaOH vs. D16 6FP	358.7	367.3	-8.667	35.72	3	3	0.3431	14
D16 NaOH vs. D4 6FP	360	548.3	-188.3	35.72	3	3	7.456	14
D16 NaOH vs. D6 6FP	360	376.3	-16.33	35.72	3	3	0.6466	14
D16 NaOH vs. D8 6FP	360	363	-3	35.72	3	3	0.1188	14
D16 NaOH vs. D16 6FP	360	367.3	-7.333	35.72	3	3	0.2903	14
D4 6FP vs. D6 6FP	548.3	376.3	172	35.72	3	3	6.809	14
D4 6FP vs. D8 6FP	548.3	363	185.3	35.72	3	3	7.337	14
D4 6FP vs. D16 6FP	548.3	367.3	181	35.72	3	3	7.166	14
D6 6FP vs. D8 6FP	376.3	363	13.33	35.72	3	3	0.5278	14
D6 6FP vs. D16 6FP	376.3	367.3	9	35.72	3	3	0.3563	14
D8 6FP vs. D16 6FP	363	367.3	-4.333	35.72	3	3	0.1716	14

Table S4: List of proteins and peptides identified by mass spectrometry after on-bead digest and DDA analysis. See separate Excel file "SupportingInformationTable_S4".

Table S5: Lists of proteins and peptides identified by mass spectrometry after in-gel digest and DIA analysis. See separate Excel file "SupportingInformationTable_S5".

Table S6: Nonlinear fit Table of results for Figure 6B. Table of Results for nonlinear regression analysis to determine whether data sets differ from each other. One phase decay models were fit using least squares regression and compared using the extra sum-of-squares F test in Graphpad Prism 10.4.1.

**Comparison of Fits**			
Null hypothesis			One curve for all data sets
Alternative hypothesis			Different curve for at least one data set
P value			0.0007
F (DFn, DFd)			9.165 (3, 18)
**Different curve for at least one data set**			
Best-fit values	D4	D6	Global
Y0	104.1	101.2	
Plateau	15.59	13.65	
K	0.01665	0.04936	
Half Life	41.62	14.04	
Tau	60.05	20.26	
Span	88.55	87.56	
95% CI (profile likelihood)			
Y0	87.99 to 120.8	94.76 to 107.7	
Plateau	??? to 44.22	6.759 to 20.27	
K	??? to 0.04246	0.03868 to 0.06289	
Half Life	16.32 to ???	11.02 to 17.92	
Tau	23.55 to ???	15.90 to 25.85	
Goodness of Fit			
Degrees of Freedom	9	9	
R squared	0.8484	0.982	
Sum of Squares	1797	243.7	
Sy.x	14.13	5.203	
Constraints			
K	K > 0	K > 0	
**One curve for all data sets**			
Best-fit values			
Y0	102.2	102.2	102.2
Plateau	18.94	18.94	18.94
K	0.03168	0.03168	0.03168
Half Life	21.88	21.88	21.88
Tau	31.57	31.57	31.57
Span	83.23	83.23	83.23
95% CI (profile likelihood)			
Y0	90.03 to 114.5	90.03 to 114.5	90.03 to 114.5

Plateau	-1.844 to 33.44	-1.844 to 33.44	-1.844 to 33.44
K	0.01528 to 0.05376	0.01528 to 0.05376	0.01528 to 0.05376
Half Life	12.89 to 45.38	12.89 to 45.38	12.89 to 45.38
Tau	18.60 to 65.46	18.60 to 65.46	18.60 to 65.46
Goodness of Fit			
Degrees of Freedom			21
R squared	0.718	0.8659	0.8127
Sum of Squares	3344	1813	5157
Sy.x			15.67
Constraints			
Y0	Y0 is shared	Y0 is shared	
Plateau	Plateau is shared	Plateau is shared	
K	K > 0 and shared	K > 0 and shared	
Number of points			
# of X values	12	12	
# Y values analyzed	12	12	

Table S7: Nonlinear fit Table of results for surface MR1 in Figure 6C. Table of Results for nonlinear regression analysis to determine whether data sets differ from each other. One phase decay models were fit using least squares regression with K constrained to > 0 and compared using the extra sum-of-squares F test in Graphpad Prism 10.4.1.

**Comparison of Fits**			
Null hypothesis			One curve for all data sets
Alternative hypothesis			Different curve for at least one data set
P value			<0.0001
F (DFn, DFd)			57.92 (3, 24)
**Different curve for at least one data set**			
Best-fit values	D4	D6	Global
Y0	100.7	100	
Plateau	3.244E-09	6.88	
K	0.117	0.3391	
Half Life	5.924	2.044	
Tau	8.546	2.949	
Span	100.7	93.13	
95% CI (profile likelihood)			
Y0	96.20 to 105.2	93.47 to 106.6	
Plateau	??? to 35.67	??? to 19.88	
K	0.09929 to 0.2295	0.2546 to 0.4889	
Half Life	3.020 to 6.981	1.418 to 2.723	
Tau	4.357 to 10.07	2.045 to 3.928	
Goodness of Fit			
Degrees of Freedom	12	12	
R squared	0.9524	0.9727	
Sum of Squares	251.4	356.5	
Sy.x	4.577	5.451	
Constraints			
Plateau	Plateau > 0	Plateau > 0	
K	K > 0	K > 0	
**One curve for all data sets**			
Best-fit values			
Y0	99.56	99.56	99.56
Plateau	9.786	9.786	9.786
K	0.2196	0.2196	0.2196
Half Life	3.156	3.156	3.156
Tau	4.553	4.553	4.553
Span	89.78	89.78	89.78
95% CI (profile likelihood)			

Y0	89.68 to 110.5	89.68 to 110.5	89.68 to 110.5
Plateau	-infinity to 40.23	-infinity to 40.23	-infinity to 40.23
K	0.1395 to 0.5252	0.1395 to 0.5252	0.1395 to 0.5252
Half Life	1.320 to 4.969	1.320 to 4.969	1.320 to 4.969
Tau	1.904 to 7.169	1.904 to 7.169	1.904 to 7.169
Goodness of Fit			
Degrees of Freedom			27
R squared	0.5371	0.8033	0.7687
Sum of Squares	2442	2567	5009
Sy.x			13.62
Constraints			
Y0	Y0 is shared	Y0 is shared	
Plateau	Plateau > 0 and shared	Plateau > 0 and shared	
K	K > 0 and shared	K > 0 and shared	
Number of points			
# of X values	15	15	
# Y values analyzed	15	15	

Table S8: Nonlinear fit Table of results for total MR1 (GFP) in Figure 6C. Table of Results for nonlinear regression analysis to determine whether data sets differ from each other. One phase decay models were fit using least squares regression with K constrained to > 0 and compared using the extra sum-of-squares F test in Graphpad Prism 10.4.1.

**Comparison of Fits**			
Null hypothesis			One curve for all data sets
Alternative hypothesis			Different curve for at least one data set
P value			<0.0001
F (DFn, DFd)			50.41 (3, 24)
**Different curve for at least one data set**			
Best-fit values			
Y0	102.2	100.5	
Plateau	2.226E-11	20.94	
K	0.08907	0.2052	
Half Life	7.782	3.378	
Tau	11.23	4.873	
Span	102.2	79.61	
95% CI (profile likelihood)			
Y0	99.78 to 104.7	97.41 to 103.7	
Plateau	??? to 29.37	??? to 35.30	
K	0.08013 to 0.1414	0.1340 to 0.2979	
Half Life	4.901 to 8.650	2.327 to 5.174	
Tau	7.070 to 12.48	3.357 to 7.464	
Goodness of Fit			
Degrees of Freedom	12	12	
R squared	0.9777	0.9858	
Sum of Squares	79.45	88.31	
Sy.x	2.573	2.713	
Constraints			
Plateau	Plateau > 0	Plateau > 0	
K	K > 0	K > 0	
**One curve for all data sets**			
Best-fit values			
Y0	100.7	100.7	100.7
Plateau	1.694	1.694	1.694
K	0.1151	0.1151	0.1151
Half Life	6.024	6.024	6.024
Tau	8.691	8.691	8.691
Span	99	99	99
95% CI (profile likelihood)			

Y0	96.30 to 105.9	96.30 to 105.9	96.30 to 105.9
Plateau	??? to 44.68	??? to 44.68	??? to 44.68
K	0.09522 to 0.2863	0.09522 to 0.2863	0.09522 to 0.2863
Half Life	2.421 to 7.279	2.421 to 7.279	2.421 to 7.279
Tau	3.493 to 10.50	3.493 to 10.50	3.493 to 10.50
Goodness of Fit			
Degrees of Freedom			27
R squared	0.8328	0.8988	0.8845
Sum of Squares	597.1	627.7	1225
Sy.x			6.735
Constraints			
Y0	Y0 is shared	Y0 is shared	
Plateau	Plateau > 0 and shared	Plateau > 0 and shared	
K	K > 0 and shared	K > 0 and shared	
Number of points			
# of X values	15	15	
# Y values analyzed	15	15	

Table S9: ANOVA table for GFP GeoMFI with dox titration. Relates to Figure 8C. Table of Results for repeated-measures one-way ANOVA with Tukey's multiple comparisons test comparing GFP GeoMFI between experimental groups in Graphpad Prism 10.4.1.

**Repeated measures ANOVA summary**					
Assume sphericity?	Yes				
F	124.6				
P value	<0.0001				
P value summary	****				
Statistically significant (P < 0.05)?	Yes				
R squared	0.9842				
Was the matching effective?					
F	39.87				
P value	<0.0001				
P value summary	****				
Is there significant matching (P < 0.05)?	Yes				
R squared	0.06543				
ANOVA table	SS	DF	MS	F (DFn, DFd)	P value
Treatment (between columns)	18377	9	2042	F (9, 18) = 124.6	P<0.0001
Individual (between rows)	1307	2	653.6	F (2, 18) = 39.87	P<0.0001
Residual (random)	295.1	18	16.39		
Total	19979	29			
Data summary					
Number of treatments (columns)	10				
Number of subjects (rows)	3				
Number of missing values	0				

Number of families			1					
Number of comparisons per family			45					
Alpha			0.05					
**Tukey's multiple comparisons test**								
	Mean Diff.	95.00% CI of diff.	Below threshold?	Summary	Adjusted P Value			
mis 0 vs. mis 62.5	-3.646	-15.50 to 8.208	No	ns	0.978			
mis 0 vs. mis 125	-11.32	-23.17 to 0.5335	No	ns	0.0682			
mis 0 vs. mis 250	-35.49	-47.34 to -23.64	Yes	****	<0.0001			
mis 0 vs. mis 500	-77.56	-89.41 to -65.71	Yes	****	<0.0001			
mis 0 vs. KD 0	-1.02	-12.87 to 10.83	No	ns	>0.9999			

mis 0 vs. KD 62.5	-2.803	-14.66 to 9.050	No	ns	0.9964			
mis 0 vs. KD 125	-8.919	-20.77 to 2.935	No	ns	0.2449			
mis 0 vs. KD 250	-23.05	-34.90 to -11.19	Yes	****	<0.0001			
mis 0 vs. KD 500	-52.7	-64.55 to -40.84	Yes	****	<0.0001			
mis 62.5 vs. mis 125	-7.674	-19.53 to 4.179	No	ns	0.4214			
mis 62.5 vs. mis 250	-31.84	-43.70 to -19.99	Yes	****	<0.0001			
mis 62.5 vs. mis 500	-73.91	-85.77 to -62.06	Yes	****	<0.0001			
mis 62.5 vs. KD 0	2.626	-9.227 to 14.48	No	ns	0.9978			
mis 62.5 vs. KD 62.5	0.8427	-11.01 to 12.70	No	ns	>0.9999			
mis 62.5 vs. KD 125	-5.273	-17.13 to 6.580	No	ns	0.8344			
mis 62.5 vs. KD 250	-19.4	-31.25 to -7.548	Yes	***	0.0005			
mis 62.5 vs. KD 500	-49.05	-60.90 to -37.20	Yes	****	<0.0001			
mis 125 vs. mis 250	-24.17	-36.02 to -12.32	Yes	****	<0.0001			
mis 125 vs. mis 500	-66.24	-78.09 to -54.39	Yes	****	<0.0001			
mis 125 vs. KD 0	10.3	-1.553 to 22.15	No	ns	0.1208			
mis 125 vs. KD 62.5	8.517	-3.336 to 20.37	No	ns	0.2951			
mis 125 vs. KD 125	2.401	-9.452 to 14.25	No	ns	0.9989			
mis 125 vs. KD 250	-11.73	-23.58 to 0.1257	No	ns	0.0538			
mis 125 vs. KD 500	-41.38	-53.23 to -29.52	Yes	****	<0.0001			
mis 250 vs. mis 500	-42.07	-53.92 to -30.22	Yes	****	<0.0001			
mis 250 vs. KD 0	34.47	22.62 to 46.32	Yes	****	<0.0001			
mis 250 vs. KD 62.5	32.69	20.83 to 44.54	Yes	****	<0.0001			
mis 250 vs. KD 125	26.57	14.72 to 38.42	Yes	****	<0.0001			
mis 250 vs. KD 250	12.44	0.5891 to 24.30	Yes	*	0.0352			
mis 250 vs. KD 500	-17.21	-29.06 to -5.355	Yes	**	0.0018			
mis 500 vs. KD 0	76.54	64.69 to 88.39	Yes	****	<0.0001			
mis 500 vs. KD 62.5	74.76	62.90 to 86.61	Yes	****	<0.0001			
mis 500 vs. KD 125	68.64	56.79 to 80.49	Yes	****	<0.0001			
mis 500 vs. KD 250	54.51	42.66 to 66.36	Yes	****	<0.0001			
mis 500 vs. KD 500	24.86	13.01 to 36.71	Yes	****	<0.0001			
KD 0 vs. KD 62.5	-1.783	-13.64 to 10.07	No	ns	0.9999			
KD 0 vs. KD 125	-7.899	-19.75 to 3.954	No	ns	0.3851			
KD 0 vs. KD 250	-22.03	-33.88 to -10.17	Yes	***	0.0001			
KD 0 vs. KD 500	-51.68	-63.53 to -39.82	Yes	****	<0.0001			
KD 62.5 vs. KD 125	-6.116	-17.97 to 5.737	No	ns	0.6989			
KD 62.5 vs. KD 250	-20.24	-32.10 to -8.391	Yes	***	0.0003			
KD 62.5 vs. KD 500	-49.89	-61.75 to -38.04	Yes	****	<0.0001			
KD 125 vs. KD 250	-14.13	-25.98 to -2.275	Yes	*	0.0126			
KD 125 vs. KD 500	-43.78	-55.63 to -31.93	Yes	****	<0.0001			

KD 250 vs. KD 500	-29.65	-41.50 to -17.80	Yes	****	<0.0001			
Test details	Mean 1	Mean 2	Mean Diff.	SE of diff.	n1	n2	q	DF
mis 0 vs. mis 62.5	22.44	26.09	-3.646	3.306	3	3	1.56	18
mis 0 vs. mis 125	22.44	33.76	-11.32	3.306	3	3	4.842	18
mis 0 vs. mis 250	22.44	57.93	-35.49	3.306	3	3	15.18	18
mis 0 vs. mis 500	22.44	100	-77.56	3.306	3	3	33.18	18
mis 0 vs. KD 0	22.44	23.46	-1.02	3.306	3	3	0.4362	18
mis 0 vs. KD 62.5	22.44	25.24	-2.803	3.306	3	3	1.199	18
mis 0 vs. KD 125	22.44	31.36	-8.919	3.306	3	3	3.815	18
mis 0 vs. KD 250	22.44	45.49	-23.05	3.306	3	3	9.859	18
mis 0 vs. KD 500	22.44	75.14	-52.7	3.306	3	3	22.54	18
mis 62.5 vs. mis 125	26.09	33.76	-7.674	3.306	3	3	3.283	18
mis 62.5 vs. mis 250	26.09	57.93	-31.84	3.306	3	3	13.62	18
mis 62.5 vs. mis 500	26.09	100	-73.91	3.306	3	3	31.62	18
mis 62.5 vs. KD 0	26.09	23.46	2.626	3.306	3	3	1.123	18
mis 62.5 vs. KD 62.5	26.09	25.24	0.8427	3.306	3	3	0.3605	18
mis 62.5 vs. KD 125	26.09	31.36	-5.273	3.306	3	3	2.256	18
mis 62.5 vs. KD 250	26.09	45.49	-19.4	3.306	3	3	8.299	18
mis 62.5 vs. KD 500	26.09	75.14	-49.05	3.306	3	3	20.98	18
mis 125 vs. mis 250	33.76	57.93	-24.17	3.306	3	3	10.34	18
mis 125 vs. mis 500	33.76	100	-66.24	3.306	3	3	28.34	18
mis 125 vs. KD 0	33.76	23.46	10.3	3.306	3	3	4.406	18
mis 125 vs. KD 62.5	33.76	25.24	8.517	3.306	3	3	3.643	18
mis 125 vs. KD 125	33.76	31.36	2.401	3.306	3	3	1.027	18
mis 125 vs. KD 250	33.76	45.49	-11.73	3.306	3	3	5.017	18
mis 125 vs. KD 500	33.76	75.14	-41.38	3.306	3	3	17.7	18
mis 250 vs. mis 500	57.93	100	-42.07	3.306	3	3	18	18
mis 250 vs. KD 0	57.93	23.46	34.47	3.306	3	3	14.75	18
mis 250 vs. KD 62.5	57.93	25.24	32.69	3.306	3	3	13.98	18
mis 250 vs. KD 125	57.93	31.36	26.57	3.306	3	3	11.37	18
mis 250 vs. KD 250	57.93	45.49	12.44	3.306	3	3	5.323	18
mis 250 vs. KD 500	57.93	75.14	-17.21	3.306	3	3	7.361	18
mis 500 vs. KD 0	100	23.46	76.54	3.306	3	3	32.74	18
mis 500 vs. KD 62.5	100	25.24	74.76	3.306	3	3	31.98	18
mis 500 vs. KD 125	100	31.36	68.64	3.306	3	3	29.36	18
mis 500 vs. KD 250	100	45.49	54.51	3.306	3	3	23.32	18
mis 500 vs. KD 500	100	75.14	24.86	3.306	3	3	10.63	18
KD 0 vs. KD 62.5	23.46	25.24	-1.783	3.306	3	3	0.7628	18

KD 0 vs. KD 125	23.46	31.36	-7.899	3.306	3	3	3.379	18
KD 0 vs. KD 250	23.46	45.49	-22.03	3.306	3	3	9.423	18
KD 0 vs. KD 500	23.46	75.14	-51.68	3.306	3	3	22.11	18
KD 62.5 vs. KD 125	25.24	31.36	-6.116	3.306	3	3	2.616	18
KD 62.5 vs. KD 250	25.24	45.49	-20.24	3.306	3	3	8.66	18
KD 62.5 vs. KD 500	25.24	75.14	-49.89	3.306	3	3	21.34	18
KD 125 vs. KD 250	31.36	45.49	-14.13	3.306	3	3	6.044	18
KD 125 vs. KD 500	31.36	75.14	-43.78	3.306	3	3	18.73	18
KD 250 vs. KD 500	45.49	75.14	-29.65	3.306	3	3	12.68	18

Table S10: Proteins identified by mass spectrometry after in-gel digest and DIA analysis that showed a statistically significant batch effect.

ProteinID	Gene	ConditionWT	BatchDay2	BatchDay3	AveExpr	F	P.Value	adj.P.Val
Q9UQ35	SRRM2	0.28528	-1.05295	-6.06925	16.35781	1654.77	3.61E-22	5.33E-19
Q92572	AP3S1	0.076903	0.522364	-3.97102	14.57861	1395.993	1.66E-21	1.22E-18
Q13144	EIF2B5	0.064008	0.943977	-5.69049	13.01635	1026.646	2.60E-20	1.28E-17
Q8NC51	SERBP1	0.022965	-0.29102	-2.72809	14.91425	963.8496	4.58E-20	1.69E-17
P52272	HNRNPM	-0.16751	-0.52085	-2.55946	15.11057	931.0728	6.24E-20	1.84E-17
P26038	MSN	-0.0166	-1.33717	-2.95219	13.89116	907.8099	7.82E-20	1.92E-17
A0A0A0MRM9	NOLC1	0.008183	-0.68494	-5.87713	15.03803	838.0488	1.60E-19	3.37E-17
Q07065	CKAP4	0.081128	-0.26723	-2.37724	14.6895	674.0332	1.12E-18	2.06E-16
O14974	PPP1R12A	-0.15354	0.241904	-2.54269	14.0712	655.8396	1.43E-18	2.34E-16
Q86UE4	MTDH	-0.15537	-0.43513	-3.09421	14.36369	642.3895	1.72E-18	2.53E-16

Supporting Information References

1. Clement, K., et al., *CRISPResso2 provides accurate and rapid genome editing sequence analysis.* Nat Biotechnol, 2019. **37**(3): p. 224-226.
